# Ultracompact and multifunctional integrated photonic platform

**DOI:** 10.1126/sciadv.adm7569

**Published:** 2024-06-19

**Authors:** Zhuochen Du, Kun Liao, Tianxiang Dai, Yufei Wang, Jinze Gao, Haiqi Huang, Huixin Qi, Yandong Li, Xiaoxiao Wang, Xinran Su, Xingyuan Wang, Yan Yang, Cuicui Lu, Xiaoyong Hu, Qihuang Gong

**Affiliations:** ^1^ State Key Laboratory for Mesoscopic Physics & Department of Physics, Collaborative Innovation Center of Quantum Matter, Beijing Academy of Quantum Information Sciences, Nano-optoelectronics Frontier Center of Ministry of Education, Peking University, Beijing 100871, China.; ^2^ School of Instrumentation and Optoelectronic Engineering, Beihang University, Beijing 100191, China.; ^3^ College of Mathematics and Physics, Beijing University of Chemical Technology, Beijing 100029, China.; ^4^ Institute of Microelectronics, Chinese Academy of Sciences, Beijing 100029, China.; ^5^ Key Laboratory of Advanced Optoelectronic Quantum Architecture and Measurements of Ministry of Education, Beijing Key Laboratory of Nanophotonics and Ultrafine Optoelectronic Systems, School of Physics, Beijing Institute of Technology, Beijing 100081, China.; ^6^ Peking University Yangtze Delta Institute of Optoelectronics, Nantong, Jiangsu 226010, China.; ^7^ Collaborative Innovation Center of Extreme Optics, Shanxi University, Taiyuan, Shanxi 030006, China.; ^8^ Hefei National Laboratory, Hefei 230088, China.

## Abstract

Realizing a multifunctional integrated photonic platform is one of the goals for future optical information processing, which usually requires large size to realize due to multiple integration challenges. Here, we realize a multifunctional integrated photonic platform with ultracompact footprint based on inverse design. The photonic platform is compact with 86 inverse designed-fixed couplers and 91 phase shifters. The footprint of each coupler is 4 μm by 2 μm, while the whole photonic platform is 3 mm by 0.2 mm—one order of magnitude smaller than previous designs. One-dimensional Floquet Su-Schrieffer-Heeger model and Aubry-André-Harper model are performed with measured fidelities of 97.90 (±0.52) % and 99.34 (±0.44) %, respectively. We also demonstrate a handwritten digits classification task with the test accuracy of 87% using on-chip training. Moreover, the scalability of this platform has been proved by demonstrating more complex computing tasks. This work provides an effective method to realize an ultrasmall integrated photonic platform.

## Teaser

An ultracompact and multifunctional photonic platform based on inverse design method is presented.

## INTRODUCTION

The integrated photonic platform, which takes photon as information carriers, plays a key role in optical information processing. With the increasing needs of information processing capacity (*1*–*3*), two basic demands have been proposed for integrated photonic platform, one is the multiple functions and the other is the ultracompact footprint (*3*). Traditional photonic platform based on photonic crystals (*4*, *5*), micro-rings (*6*, *7*), or metasurfaces (*8*, *9*) has been usually used to realize photonic information processing due to the unique properties of photonic bandgap, resonance response, or phase modulation, respectively. However, they suffer from a relatively large footprint, and it is difficult to realize multiple functions by directly extending the same unit structures. Photonic platforms based on surface plasmon polaritons (*10*, *11*) have a small footprint with a subwavelength scale, but it is difficult to overcome the intrinsic loss of metal to realize large-scale integration.

The Mach-Zehnder interferometer (MZI)–based platform is one of the most promising and reliable on-chip optical network structures for large-scale integration. MZI-based platforms have already been used to realize self-configuration (*12*), mode mixing (*13*, *14*), quantum simulations [Boson sampling; (*15*–*18*)], quantum transport simulation (*19*, *20*), quantum walk simulation (*21*–*23*), molecular simulation (*24*–*27*), quantum gates (*28*–*31*), quantum information processing (*32*–*35*), optical neural networks (*36*–*40*), etc. However, this platform requires a large size because the footprint of basic devices is in the order of hundreds square microns (μm^2^) and facing the challenge of increasing computing power per unit area. It is currently a great challenge to realize a large-scale multifunctional and ultracompact integrated photonic platform simultaneously.

**Fig. 1.**
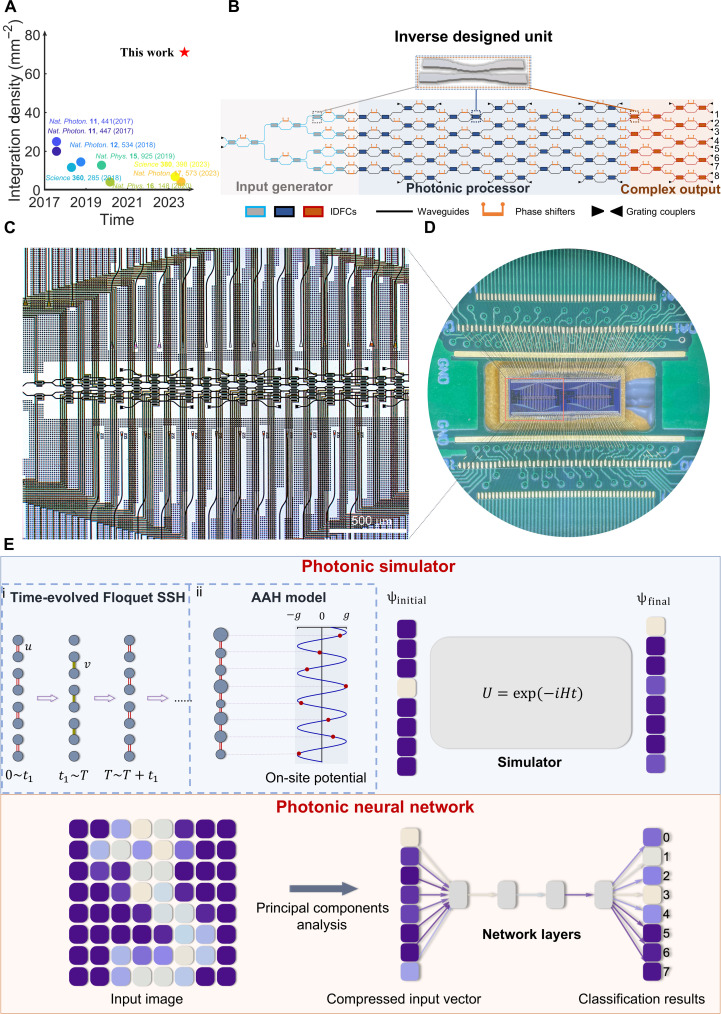
The scheme of proposed computing platform. (**A**) Comparison of integration density between our work and other MZI-based photonic platform. (**B**) Schematic diagram of the computing platform, consist of input generator, photonic processor, and complex output. The total platform has 86 IDFCs and 91 PSs to form 43 MZIS. Different areas of color denote different functions with the same units. The input generator has 14 IDFCs and 15 PSs to form seven MZIs and completely generate the input state. Photonic processor has 54 IDFCs and 63 PSs to form 27 MZIs. The complex output has 18 IDFCs and 21PSs to form nine MZIs. The final part provides the ability to measure the output intensity and relative phase. It can be merged into the photonic processor part if the phase information is undesired. (**C**) Optical microscope image of photonic platform. Scale bar, 500 μm. (**D**) Photograph of the photonic platform and wire bonding. The red square is one platform details in (C). Each chip consists of two individual computing platform. (**E**) Schematic diagrams of our experimentally realized tasks. The photonic simulator realizes quantum simulation for (i) Floquet time-evolved SSH and (ii) AAH model. The initial state is generated, and the final state is calculated by the simulator. The PNN realizes a handwritten numbers classification task. The input images are compressed after PCA and get the classification results after the network layers.

Here, we report a scheme to realize a multifunctional large-scale integrated photonic platform with an ultracompact footprint based on inverse design. An inverse designed algorithm has been developed by combining an adjoint gradient algorithm and a geometric restriction algorithm so that a high-performance inverse designed-fixed coupler (IDFC) with a footprint of only 4 μm by 2 μm is realized. The IDFC’s insertion loss and interference visibility are measured to be −0.48 dB and 0.996, respectively. A multifunctional photonic platform includes 86 IDFCs and 91 phase shifters (PSs) with a footprint of only 3 mm by 0.2 mm. The integration density increased by one order compared to the reported MZI-based works (Fig. 1A). An overcomplete electrode distribution is adopted so that the phase shifting of each waveguide can be adjusted independently. Therefore, arbitrary form of a one-dimensional (1D) tightly binding model quantum simulation can be performed. Quantum state evolutions are performed, which include 1D Floquet Su-Schrieffer-Heeger (SSH) model and 1D Aubry-André-Harper (AAH) model. As for the Floquet SSH model, the Hamiltonian varies periodically in time domain and has four topological phases, and the measured average fidelity is 97.90 (±0.52) %. Meanwhile, the measured average fidelity for AAH model is 99.34 (±0.44) %. We also demonstrate handwritten digits classification of four-class and eight-class tasks using on-chip training on this platform with measured test set accuracy of 87 and 60%, respectively. For the input handwritten images, the principal components analysis (PCA) dimensionality reduction algorithm is used in advance, so that the information can be encoded into the photonic platform. Furthermore, the scalability of the photonic platform is demonstrated by calculating 2D quantum simulation for disorder SSH model and 10-class classification task. This work makes a breakthrough by providing a feasible solution for implementation of a large-scale, ultracompact, and multifunctional photonic platform based on inverse design, promoting the integration density by one order, which is applicable for any other photonic platform configuration.

## RESULTS

### Scheme of photonic platform

Figure 1B shows the diagram of the computing platform, which consist of input generator, photonic processor, and complex output. The input generator has 14 IDFCs and 15 PSs to form seven MZIs. Three columns of PSs on the left completely realize the generation of amplitude. The fourth column can adjust the phase of all channels to completely build the input states. The photonic processor consists of 54 IDFCs and 63 PSs to form 27 MZIs. Note that the first column of PSs is shared with input generator. The transfer matrix of the odd column can be represented as

$$U_{1}={⊕}_{i=1}^{4}U_{0}\left(\alpha_{i},\beta_{i},\gamma_{i},\delta_{i}\right)$$ (1)

$$U_{0}\left(\alpha_{i},\beta_{i},\gamma_{i},\delta_{i}\right)=e^{i\delta_{i}}\left[\begin{array}{cc} e^{i\beta_{i}}\text{cos}\alpha_{i} & e^{-i\gamma_{i}}\text{sin}\alpha_{i}\\ -e^{i\gamma_{i}}\text{sin}\alpha_{i} & e^{-i\beta_{i}}\text{cos}\alpha_{i} \end{array}\right]$$ (2)

Here, α*_i_* ∈ [0, π), β*_i_* ∈ [0,2π), γ*_i_* ∈ [0,2π), δ*_i_* ∈ [0,2π), they represent the four parameters of second-order unitary matrices. *U*_0_(α*_i_*, β*_i_*, γ*_i_*, δ*_i_*) completely constructs all the two-order unitary matrices between the 2*i* − 1 and 2*i* waveguide. Here, the even column can be represented as

$$U_{2}={⊕}_{j=1}^{3}U_{0}\left(\alpha_{j},\beta_{j},\gamma_{j},\delta_{j}\right)$$ (3)

*U*_0_(α*_j_*, β*_j_*, γ*_j_*, δ*_j_*) forms the two-order unitary matrices between the 2*j* and 2*j* + 1 waveguide, while the first and eighth waveguide remain identical. The total transfer matrix of this photonic processor is

$$U=\prod_{i=1}^{3}U_{2}^{\left(i\right)}U_{1}^{\left(i\right)}$$ (4)

The complex output has the ability to measure both output intensity and relative phase between waveguides (see note S2). Note that the complex output part can be merged into the photonic processor part if the phase information is undesired for the specific task.

The optical microscope image of photonic platform is shown in Fig. 1C. The scale bar is 500 μm. Figure 1D is the image of the whole well-packaged photonic platform with wire bonding. The area enclosed in red corresponds to one platform, which is detailed in Fig. 1C. Figure 1E shows the schematic diagrams of our experimentally realized tasks. First, the photonic platform performs as a simulator to realize quantum simulation for (i) Floquet time-evolved SSH and (ii) AAH model. The initial state is generated by the input generator. The photonic processor performs the simulation, and the final states can be measured. Then, we regard the photonic platform as a photonic neural network (PNN) capable of on-chip training to realize the handwritten digits classification task. Here, the schematic diagram shows an eight-class classification task as an example. The input images are compressed after PCA, and we can get the classification results after the network layers.

As we can see from the schematic diagram of the inverse designed 2 × 2 fixed coupler in inset of Fig. 1B. Different from the traditional multi-mode interferometer (MMI) structure, our IDFC adopts asymmetric configuration. The transfer matrix of the IDFC is

$$F_{\text{IDFC}}=\frac{1}{\sqrt{2}}\left[\begin{array}{cc} 1 & 1\\ 1 & -1 \end{array}\right]$$ (5)

The cross-section size of input or output waveguides is 500 nm by 220 nm. The footprint of the IDFC is 4 μm by 2 μm, which is tens of times less than the size of the traditional MMIs. The two waveguides at the same side are separated by 500 nm to avoid unwanted cross-talk.

The inverse designed device is equally divided into 200 × 100 identical units in the designing process, the size of each unit is 20 nm by 20 nm by 220 nm. We use MATLAB to control optimization process and use finite difference time domain (FDTD) software Lumerical FDTD Solutions to simulate the electromagnetic field distribution. The design process consists of two parts: the continuous optimization stage and the discrete optimization stage. At the continuous optimization stage, each unit’s refractive index changes continuously between the lower (1.444) and upper (3.476) bound. While in the discrete stage, the refractive index of units is forced to be 1.444 or 3.476 to satisfy the real condition of the material. We define an objective function to describe the performance of the device and use gradient descent algorithm to optimize the objective function. More details can be found in Materials and Methods. Here, we use the fundamental transverse electric mode of the waveguide propagating in waveguide.

**Fig. 2.**
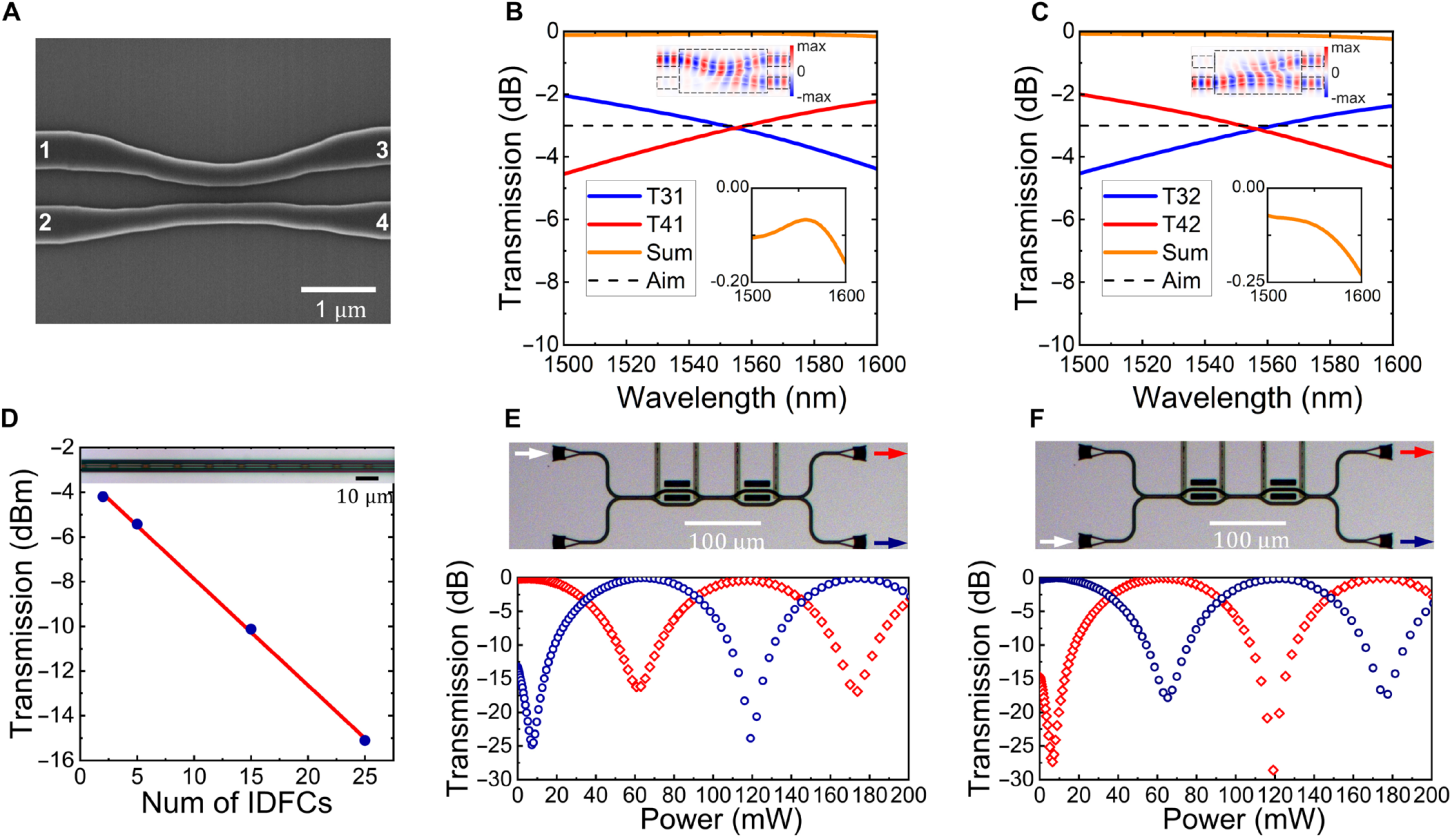
Characterization of key features of the IDFC. (**A**) SEM image of IDFC. Scale bar, 1 μm. (**B** and **C**) Simulated transmission spectra for upper and lower port input, correspondingly. The blue solid lines refer to the upper port output, while the red solid lines refer to the lower port output. The black dashed lines are the aim transmission, which is −3 dB. The orange solid lines are the total transmission of the output port and detailed in the inset below. The upper inset is the simulated real(*E_y_*) distribution at 1550-nm wavelength. (**D**) Experimentally measured insertion loss result. The measured transmission at 1550-nm wavelength with 2, 5, 15, and 25 cascaded IDFCs. The transmission in log scale has a linear relationship with the number of IDFCs. The inset shows the optical microscope image of cascaded IDFCs. (**E** and **F**) Experimental measured test MZI’s transmission versus electric power of PSs. The red points are upper output, and the blue points are lower output. The inset shows the optical microscope image of test MZI and schematic input and output arrow.

The scanning electron microscope (SEM) of the IDFC is shown in Fig. 2A, which almost fits the designed structure. However, because of the fabrication error and limited accuracy, the experimental result cannot be the same with simulation result. The robustness of inverse design guarantees that the results are disturbance rejection against the error. Figure 2 (B and C) shows the simulated transfer spectra with upper and lower port input. The *S_ij_*, refers to *j*th port input and *i*th port output. The port’s indexes are labeled in Fig. 2A. The dotted line is the ideal transmission, which is −3 dB. The orange line is the total transmission of the output port detailed in the inset. The losses at 1550 nm are lower than 0.1 dB, which are lower than that of the traditionally designed MMI. The distribution of real(*E_y_*) are shown in the inset, correspondingly. The lower port input case shows a π-phase shift between the two output lights, which fits our objective. The simulated results indicate the good performance of our designed device.

We have experimentally measured the performances of our proposed IDFC. As shown in Fig. 2D, the insertion loss of IDFC is measured to be −0.48 dB per unit device by fitting the transmission rate in log scale. This is of the same order to the results of traditional MMI, indicating the scalability of our IDFC. The measured insertion loss is larger than the simulation result, which may attribute to the fabrication error. The visibility of IDFC is also measured in experiment by connecting two IDFCs and one PSs to observe the MZI’s output intensity. Figure 2 (E and F) shows the transmission with the increasing of bias. The averaged measured visibility is 0.996, and π power is 58.6 mW, which demonstrates the good performance of IDFC.

### Quantum simulation for 1D topological model

The computation for a quantum system is a widely known problem because the dimension of Hilbert space grows exponentially with the particles numbers (*41*). A potential solution is quantum simulation, whose definition is “the computer itself be built of quantum mechanical elements, which obey quantum mechanical laws” (*41*). On-chip photonic quantum simulation is a promising platform due to its low computational overhead and fast computation speed. Theoretically, our platform can simulate most of 1D tight-binding models (TBMs) Hamiltonian although time dependent. The Hamiltonian can be written as

$$\begin{array}{c} H=\sum_{i=1}^{N}\varepsilon_{i}c_{i}^{†}c_{i}+(\sum_{i=1}^{N-1}\kappa_{i}e^{i\phi_{i}}c_{i+1}^{†}c_{i}+h.c.)=H_{1}+H_{2}\\ =[\sum_{i=1,3,5\ldots }\varepsilon_{i}c_{i}^{†}c_{i}+(\kappa_{i}e^{i\phi_{i}}c_{i+1}^{†}c_{i}+h.c.)]\\ +[\sum_{i=2,4,6\ldots }\varepsilon_{i}c_{i}^{†}c_{i}+(\kappa_{i}e^{i\phi_{i}}c_{i+1}^{†}c_{i}+h.c.)] \end{array}$$ (6)

Here, ε*_i_* is the on-site energy for the *i*th site, and $a_{i}\left(a_{i}^{†}\right)$ is the annihilation (creation) operator. κ*_i_* is the hopping coefficient, and ϕ*_i_* is the phase. The evolution operator is

$$U\left(t\right)=T\text{exp}\;\left(-i\int_{0}^{t}Hdt\prime \right)$$ (7)

where *T* represents time-ordering operator. In simulation, we use transfer matrix to map evolution operator *U*(*t*), and we use Trotter equations (*42*)

$$\begin{array}{c} U\left(t\right)=\prod \text{exp}\left(-iH_{1}\Delta t\right)\text{exp}\left(-iH_{2}\Delta t\right)+O\left[{\left(\Delta t\right)}^{2}\right]\\ =\prod \text{exp}\left(-\frac{iH_{1}\Delta t}{2}\right)\text{exp}\left(-iH_{2}\Delta t\right)\text{exp}\left(-\frac{iH_{1}\Delta t}{2}\right)+O\left[{\left(\Delta t\right)}^{3}\right] \end{array}$$ (8)

where Δ*t* represents the time step. The first line and second line are the first- and second-order Trotter equations, respectively. We can adjust the PSs (see the Supplementary Materials for details) to realize

$$U_{1}=\text{exp}\left(-iH_{1}\Delta t\right)\;U_{2}=\text{exp}\left(-iH_{2}\Delta t\right)$$ (9)

**Fig. 3.**
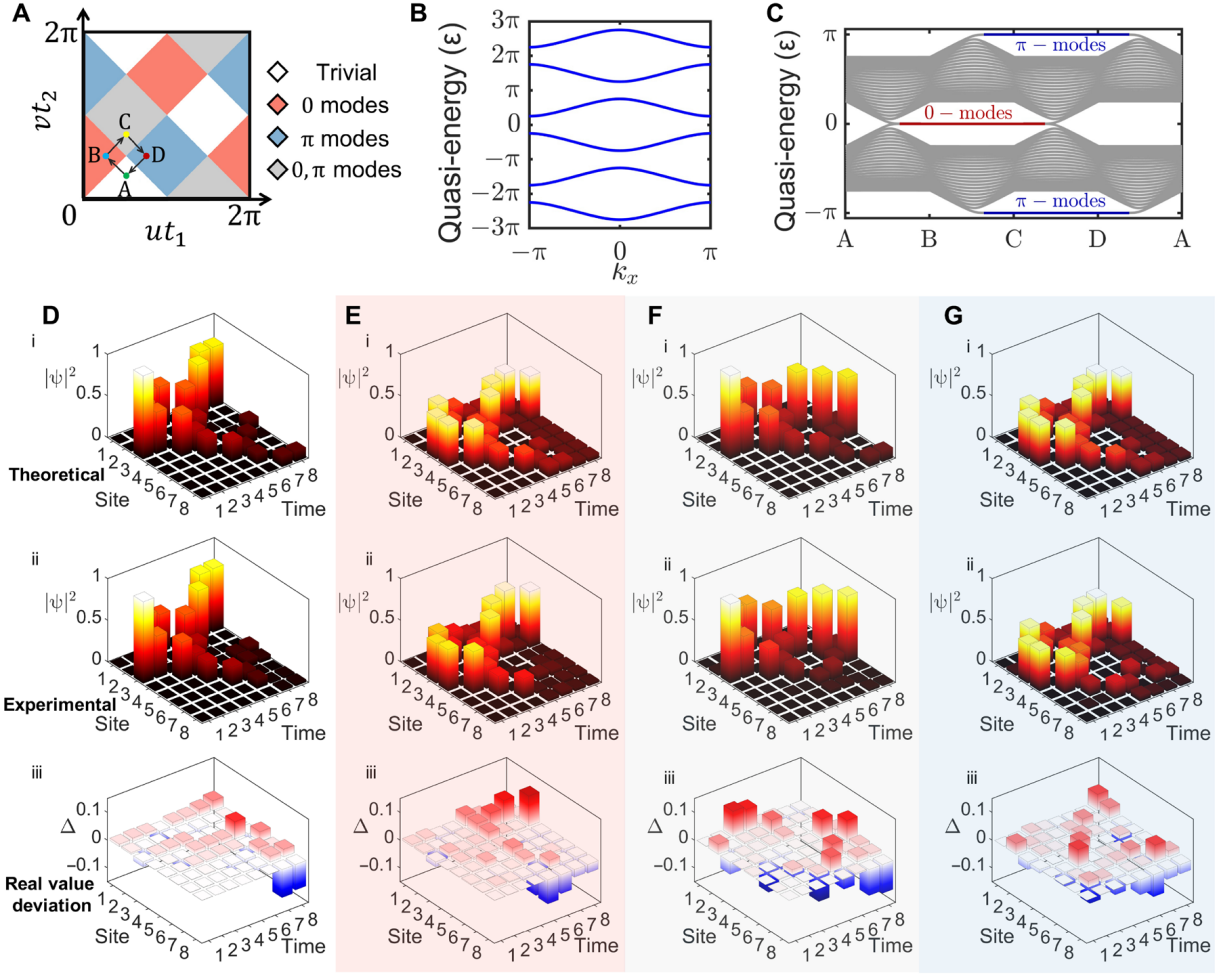
Results for Floquet-evolved SSH model. (**A**) Topological phase diagram for Floquet-evolved SSH model. The white, red, blue, and gray part represent trivial phase, topological π-mode phase and topological 0,π-mode phase. The A, B, C, and D represent four parameters in the following experiments, $(i)u=\frac{\pi }{2},v=\frac{\pi }{4},\left(\text{ii}\right)\;u=\frac{\pi }{4},v=\frac{\pi }{2},\left(\text{iii}\right)\;u=\frac{\pi }{2},v=\frac{3\pi }{4},\text{and}\left(\text{iv}\right)\;u=\frac{3\pi }{4},v=\frac{\pi }{2}$ . (**B**) PBC quasi-energy band for Floquet-evolved SSH model. The PBC band has the same form at A, B, C, and D. The band is periodic in both the quasi-momentum and quasi-energy. This figure shows three complete periods of quasi-energy. (**C**) OBC quasi-energy band along the path of A-B-C-D-A. The appearance of edge states is consistent with the topological phase diagram. (**D** to **G**) Theoretical, experimental, and deviation results of quantum simulation, corresponding to the parameters at A, B, C, and D. The *x* axis represents evolution time, *y* axis represents number of site, and *z* axis is ∣ψ∣^2^. The initial state is ∣ψ(0)⟩ = ∣2, *B*⟩ = ∣ 4⟩. The deviation results show the differences between the experimental results and theoretical results.

As one of the most important models in solid state physics, the 1D TBM can be used to describe many physical phenomena. Among these, topological insulators (TIs) have attracted widespread attention. SSH model (*43*) is one of the most well-known 1D TIs, which describes spinless fermions on an 1D lattice with the alternating distributed hopping coefficients, and supports a topological trivial and nontrivial phase. The TIs will appear more topological phase (see Fig. 3A) by introducing periodic modulation in time domain, which is also called Floquet TIs (FTIs) (*44*). In this case, energy is not a good quantum number because of the time dependent of Hamiltonian. The Floquet theory is introduced to solve the quasi-energy of the system. Here, we consider the evolution operator in a whole period

$$U\left(T\right)=T\text{exp}\;\left(-i\int_{0}^{T}Hdt\prime \right)$$ (10)

The eigenvalues of *U*(*T*) can be represented as exp(−*i*ε*T*), where ε is so-called quasi-energy (*44*). The FTI is hard to realize in an electron system. Early works have realized FTI in photonic TIs but have relatively large volume and also nonadjustable.

Here, we realize 1D Floquet SSH model on the photonic platform. The period-varying Hamiltonian is

$$H=\{\begin{array}{cc} \sum_{i}uc_{i,A}^{†}c_{i,B}+h.c. & n(t_{1}+t_{2})<t<n(t_{1}+t_{2})+t_{1}\\ \sum_{i}vc_{i,A}^{†}c_{i+1,B}+h.c. & n\left(t_{1}+t_{2}\right)+t_{1}<t<\left(n+1\right)\left(t_{1}+t_{2}\right) \end{array}$$ (11)

where *u*(*v*) is the intracell (intercell) coupling coefficient; *t*_1_ and *t*_2_ are action time of intra- and intercoupling; and *n* = 0,1,2… is the number of periods; the two sites in one cell are labeled A and B; $c_{i,A}^{†}\left(c_{i,A}\right)$ and $c_{i,B}^{†}\left(c_{i,B}\right)$ are the annihilation (creation) operator for electron on sites A and B in the *i*th cell, correspondingly. As shown in Fig. 3A, the Floquet-evolved SSH model has four topological phases, the white, red, blue, and gray part represent trivial phase, topological π-mode phase, and topological 0,π mode phase, correspondingly. We select one parameter from each topological phase to perform photonic quantum simulation in later experiments. Here, we set *t*_1_ = *t*_2_ = 1 and A) $u=\frac{\pi }{2},v=\frac{\pi }{4}$ , B) $u=\frac{\pi }{4},v=\frac{\pi }{2}$ , C) $u=\frac{\pi }{2},v=\frac{3\pi }{4}$ , and D) $u=\frac{3\pi }{4},v=\frac{\pi }{2}$ as examples. The calculated quasi-energy band under period boundary condition (PBC) is shown in Fig. 3B. Note that the PBC quasi-energy band is all the same to A, B, C, and D. However, the open boundary condition (OBC) quasi-energy band shows different properties, which is shown in Fig. 3C. The abscissa represents the parameters change along the path A-B-C-D-A shown in Fig. 3A. Topological 0-modes and π-modes appear alternately.

The initial state is set to be ∣ψ(0)⟩ = ∣2, *B*⟩ in the experiment. The theoretical and experimental results are shown in Fig. 3 (D to G). The *x* axis represents the evolution time; the *y* axis represents the number of sites, 1 to 8 corresponding to (1, A), (1, B) … (4, B); and the *z* axis represents the ∣ψ(*t*)∣^2^ at each site. The averaged measured fidelity is 97.90 (±0.52) %, which demonstrates the high accuracy of photonic quantum simulation on this platform. Under periodic boundary conditions, the existence of spatial translational symmetry ensures that quasi-momentum commutes with the Hamiltonian. As a result, quasi-momentum can be used to characterize the energy bands. The quasi-energy can be mediately measured (see note S10).

More topological phenomena will emerge by increasing the dimensional of TI, such as higher-order topological states (*45*) and transportation of topological edge state (TES) (*46*). The available dimension of the system can be extended by introducing synthetic dimension. As an example, the 1D AAH model (*47*, *48*) describes a 2D Chern insulator, whose topological invariant is Chern number. The Hamiltonian for AAH model is

$$H=\sum_{i=1}^{N}\Omega_{i}c_{i}^{†}c_{i}+\sum_{i=1}^{N-1}\left(\kappa c_{i+1}^{†}c_{i}+h.c.\right)$$ (12)

**Fig. 4.**
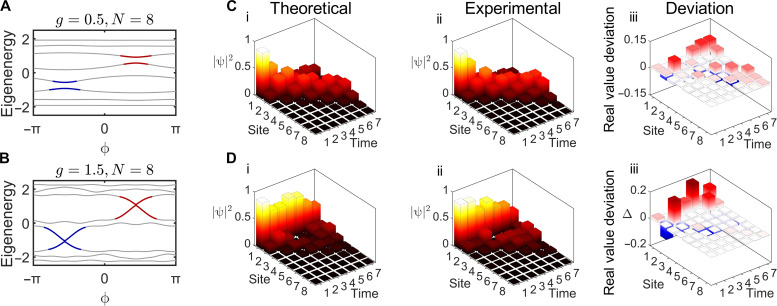
Results for AAH model. (**A** and **B**) Energy band by regarding ϕ as synthetic dimension for AAH model with *g*/κ = 0.5, *N* = 8 and *g*/κ = 1.5, *N* = 8. Edge states emerge in the gap when *g*/κ = 1.5, labeled by red and blue lines. (**C** and **D**) Theoretical, experimental, and deviation results of quantum simulation, corresponding to *g*/κ = 0.5 and 1.5. The initial state is ∣ψ(0)⟩ = ∣1⟩. For *g*/κ = 0.5, the wave packet spreads to bulk, while *g*/κ = 1.5, the wave packet propagates along the boundary.

Ω*_i_* = *g*cos(2πβ*i* + ϕ) is the on-site potential. κ is the hopping coefficient between the *i*th and (*i* + 1)th site. ϕ is the synthetic dimension. Throughout this work, we assume that $\beta =\left(\sqrt{5}-1\right)/2$ , ϕ = π/2, *g*/κ = 0.5 or 1.5, and κ = 0.4. The calculated PBC energy bands are shown in Fig. 4 (A and C), corresponding to *g*/κ = 0.5 and 1.5. In Fig. 4A, it shows a bandgap in the red and blue area. While the gap nearly closes for *g*/κ = 1.5. However, the simulation for AAH model requires each on-site potential controlled individually, which is difficult to realize. One possible solution is to move the cosine modulation to hopping coefficient term (generalized AAH model) (*49*). Therefore, a real AAH model with individually controlled on-site potential still remains a challenge to realize.

Here, we adopt an overcomplete PS distribution and construct a completely *U*(2) group in each unit to individually control the on-site potentials. The second-order Trotter equation (detailed in note S6) is used in the simulation process. The time step is set to be Δ*t* = 1 for concise illustration. The theoretical, experimental, and deviation results are shown in Fig. 4 (B and D, respectively). For *g*/*t* = 0.5, the gap is unclosed and the boundary initial state diffuses into bulk area as shown in the results. On the other hand, the gap closed for *g*/*t* = 1.5, and the state populates at the boundary in the propagating process. The average measured fidelity is 99.34 (±0.44) %. These results confirmed out that the proposed platform can simulate the 1D TBM with individual adjustable on-site potential, which is difficult for the existing platform, and greatly enrich the processable simulation tasks.

### Photonic neural network

Similar with quantum simulations, neural networks are computationally expensive. PNN provides a platform to realize high efficient computation. So far, most PNNs have been trained on computers and then transplanted the trained weights onto photonic hardware to measure the performance of the test set of the inference task. However, accurately copying weights from a computer and pasting them one-by-one onto photonic hardware is difficult due to uncertainties such as fabrication errors, large-scale devices uniformity, and modulation accuracy. Therefore, it is necessary to use on-chip training to make the network insensitive to fabrication errors and keep stable performance, thus realizing more practical, faster computing speed, and lower energy consumption large-scale PNNs. In addition to the quantum simulation task, the proposed integrated photonic platform is also capable of conducting on-chip training of PNN. The first 15 modulators can be used as a generalized input modulation, when combined with the input intensity control, allows for controlling eight complex-valued input to the network. The rest of the system functions as a PNN with four layers. We have evaluated the network performance and compared the results with simulated training on a computer with two critical tasks: a four-class handwritten image classification and an eight-class classification task to showcase the versatility and scalability of our PNN. Notably, while there are studies using diffractive neural networks for similar tasks (*50*, *51*), they are not directly comparable to our work due to the limitations in augmenting network capacity within integrated photonics. Moreover, existing research using integrated photonics often lacks the complexity required for tasks like handwritten digit classification (*40*). Our approach uniquely integrates the capability of classifying handwritten digits directly on a photonic platform without relying on digital processing. To our knowledge, this study is the first to demonstrate handwritten digit classification using an entirely integrated photonic approach, establishing a promising path in the field of integrated photonics for complex computational tasks.

The input for our PNN consists of 8 × 8–pixel handwritten images, taken from the Optical Recognition of Handwritten Digits dataset (*52*). The four-class problem corresponds to the digits 0, 1, 2, and 3, and the eight-class problem corresponds to the digits 0, 1, 2, 3, 4, 5, 6, and 7. We have 1334 training images, 200 validation images, and 720 testing images for the four-class problem, and 2861 training images, 200 validation images, and 1443 testing images for the eight-class problem. Before feeding them into the PNN, we use the PCA to reduce the dimensionality of the data from 64 to 8 while retaining most of the data variance. This preprocessing step helps in fitting the input scale to our optical neural network. We encode the processed images in the complex domain, specifically using only the real part. By encoding the input data in this manner, we are capable of taking full advantage of the complex amplitude modulation capabilities inherent in optical systems.

The final output from the PNN is measured in terms of intensity across the eight output channels. The intensity values are then used to determine the classification results, where a higher intensity corresponds to a higher probability for a particular class. For the four-class problem, the output of neighboring channels is summed to get the final results, e.g., channels 1 and 2 both determine the probability for class 1.

To conduct on-chip training on the PNN, we measured the gradients of the output to each of the input using finite difference method. We change the voltage added on the modulators by a small value and measure the corresponding change to the output. The gradient of each weight is obtained and used for gradient descent training of the PNN.

For comparison, we have implemented a simulated model for the PNN using PyTorch, which we have conducted training using the same dataset, initialization, and input data order. The gradient is computed using the same method.

**Fig. 5.**
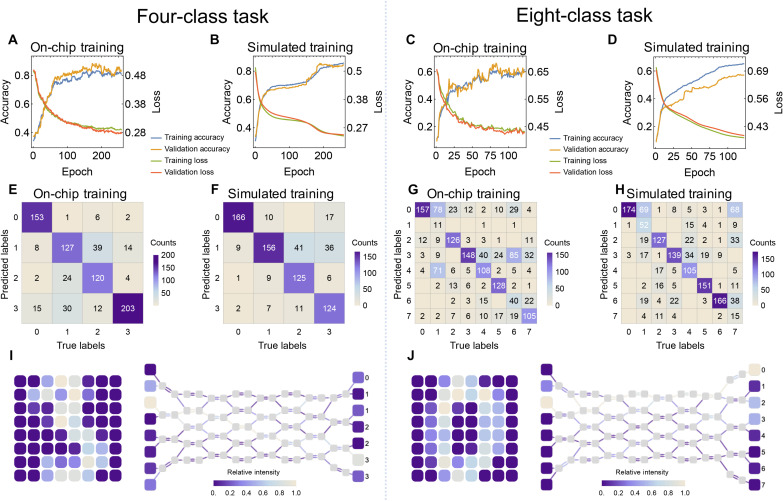
Results for handwritten digit classification. (**A** and **B**) On-chip and simulated training loss and accuracy curve of four-class problem. The loss and accuracy are measured on both training and validation sets. (**C** and **D**) On-chip and simulated training loss and accuracy curve of eight-class problem. (**E** and **F**) Confusion matrix of four-class task measured on the test set using the final model from simulated and on-chip training. (**G** and **H**) Confusion matrix of eight-class task measured on the test set using the final model from simulated and on-chip training. (**I**) Example input (digit “3”) and corresponding simulated intensity distribution in waveguides for the four-class optical neural network. The loss and accuracy are measured on both training and validation sets. (**J**) Example input (digit “0”) and corresponding simulated intensity distribution in waveguides for the eight-class optical neural network.

We have trained 260 epochs on the four-class task and 123 epochs on the eight-class task. Loss curves and accuracy curves are shown in Fig. 5 (A to D). The loss values are different by a fixed ratio due to the difference of input intensity. After training, the confusion matrix is measured on the test dataset, shown in Fig. 5 (E to H). The comparison of the on-chip and simulated training shows consistent accuracy and training patterns, suggesting that our PNN is capable of performing the tasks. The four-class and eight-class classification tasks with on-chip training are realized with test set accuracy of 87 and 60%, respectively, in comparison with training on the computer with the test set accuracy of 76 and 69%. The difference in the accuracy from the simulation indicates the random noises in the system during the training process not modeled in the simulation. For simpler tasks such as the four-class classification, noise might be less detrimental or even beneficial, enhancing signal processing and potentially leading to better performance compared to the noise-free simulation. On the contrary, for more complex tasks such as the eight-class classification, the adverse effects of noise likely outweigh these benefits, resulting in reduced accuracy. The accuracy of this network is lower in comparison to neural networks trained in computers normally used on these datasets (*53*), which is due to our network containing much fewer channels and learnable weights (91 in total) than the artificial neural networks proposed (61,706 for LeNet-5). Here, we visualize the simulated intensity distribution of light inside the waveguides for a typical input data. The input light with intensities representing input data is focused to the correct class output inside the network, as shown in Fig. 5 (I and J).

### Scalability of the proposed platform

The proposed computing platform based on inverse design can be easily extended to a larger scale. Here, we consider an extended large-scale platform with 64-in and 64-out waveguides. According to the simulation results, the theoretical insertion loss is less than 12.8 dB, which ensures a valid measured signal. Therefore, more complex tasks can be realized on this extended platform.

**Fig. 6.**
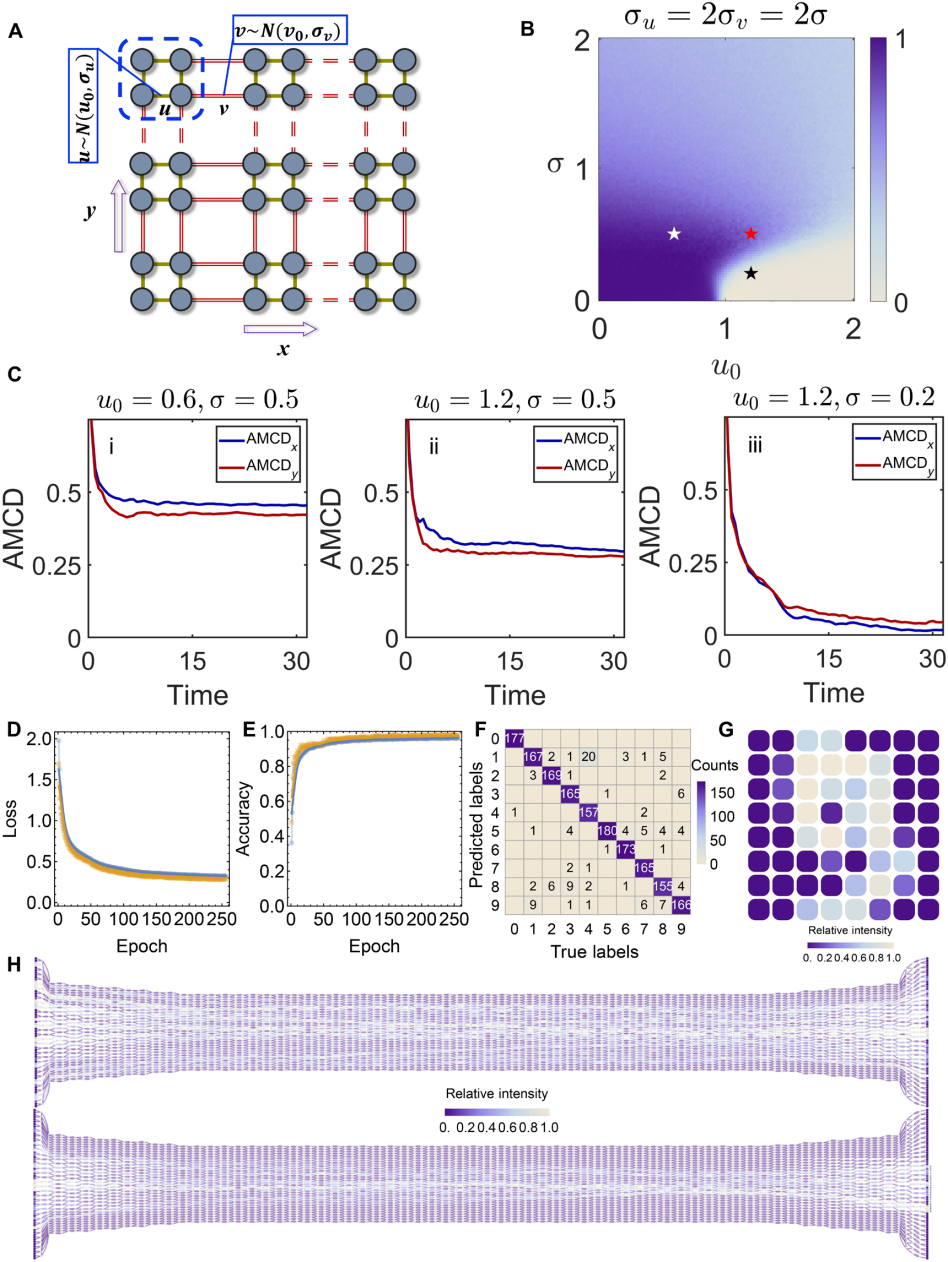
Results for scalability. (**A**) Scheme for 2D disorder SSH model. The model is based on 2D SSH model. The intracell hopping and intercell hopping obey normal distribution. (**B**) Topological phase diagram by choosing AMCD as criteria. Here, we choose *v*_0_ = 1, σ*_u_* = 2σ*_v_* = 2σ. For simplicity, we plot 2 ×AMCD here, which is the winding number. Three points labeled by white, red, and black star are chosen to perform simulation and calculate the AMCD. The results are averaged after 1000 times calculation. (**C**) Calculated AMCD for (i) *u* = 0.6, σ = 0.5, (ii) *u* = 1.2, σ = 0.5, and (iii) *u* = 1.2, σ = 0.2 after 100 times simulation. (**D**) Training loss curve of scaled model using simulation on full 10-class dataset. (**E**) Training accuracy curve of scaled model using simulation. (**F**) Confusion matrix of 10-class task measured on the test set using the final model from simulation. (**G**) Example input (digit 9) and corresponding simulated intensity distribution in waveguides for the scaled optical neural network. (**H**) Visualization of the flow of intensity for the larger network.

As an example, we theoretically realize a quantum simulation for 2D disordered SSH model (*54*). The 2D SSH model describes hopping on a 2D lattice with staggered hopping amplitudes, where each unit cell has four vertexes, and the intracellular amplitudes are *u* and intercellular amplitudes are *v*, as shown in Fig. 6A. The 2D SSH model manifests topological phases when $|\frac{u}{v}|<1$ . We denote the Hamiltonian of the 2D SSH model as *H*_2D_. Here, we have

$$H_{2D}=I_{x}⊗H_{1D}^{y}+H_{1D}^{x}⊗I_{y}$$ (13)

$$\;H_{1D}^{x}=H_{1D}^{y}=\sum_{m}u\left(c_{m,A}^{†}c_{m,B}+h.c.\right)+\sum_{m\prime }v\left(c_{m\prime ,B}^{†}c_{m\prime +1,A}+h.c.\right)$$ (14)

where *I_x_* and *I_y_* are identity operators, $c_{m,\alpha }^{†}$ and *c*_*m*,α_ represent the creation and annihilation operators of spinless fermions at the vertex α(α = *A*, *B*) in the *m*th unit cell of the SSH model. Here, we consider that the hopping amplitudes obey the normal distribution, *u*~*N*(*u*_0_, σ*_u_*) and *v*~*N*(*v*_0_, σ*_v_*). *u*_0_(*v*_0_) is the mean value and σ*_u_*(σ*_v_*) is the SD. To simplify, we set *v*_0_ = 1. We choose an averaged mean chiral displacement (AMCD) (*54*, *55*) as a criterion (see note S9) and plotting the topological phase diagram. Figure 6B shows the simulated topological phase diagram in the case σ*_u_* = 2σ*_v_*. Disorder-induced topological phase transition is observed in the trivial phase. Moreover, we also simulate the evolution of AMCD on the platform. We consider the platform uniformly divided into the lower part and the upper part. Each part consists 32 waveguides to form 16 complete lattices. An entangled photon pair is input into the platform, the state of the photon pair can be written as ∣ψ⟩ = ∣ψ_1_⟩ ⊗ ∣ψ_2_⟩. The initial state is ∣ψ(0)⟩ = ∣4, *B*⟩ ⊗ ∣4, *B*⟩ = ∣8⟩ ⊗ ∣8⟩. The simulated AMCD’s evolution for the labeled points are shown in Fig. 6 (C to E), correspondingly. The results are averaged after 100 times simulations. Simulated results of other parameters also shown in note S9. Compared with the system using the synthetic dimension in frequency dimension (*56*, *57*), this work in real space is based on the integrated silicon on-chip photonic system, while the synthetic dimension in frequency dimension is mainly realized in fiber-optical system. Both of the systems have good behaviors but they have different characteristics.

Furthermore, the extended platform can improve the performance of the PNN. We assume that by using more learnable weights in a scaled-up structure with multiple stacked optical nonlinear layers and large-scale cascaded waveguides, optical neural networks may achieve similar performance as the conventional neural networks on computers. To confirm this assumption, we demonstrate a scaled-up version of the platform with similar design patterns using a simulation. The simulated network contains 64 input channels and output channels, with 32 stacks of waveguides in each block compared to the four stacks in the experimental design. In this design, each block contains 3168 weights. With this amount of input channels, we are able to remove the PCA and input the image directly as a flattened vector. For the full network, we use two blocks, measuring the output intensity of the first block and using it as the input to the second block. We simulated the training of this network with the same setup using mean squared error as the loss function for 256 epochs, shown in Fig. 6D, achieving a training and validation accuracy of more than 95% (Fig. 6E). We also measured the confusion matrix on the test dataset, with a final accuracy of 94%, as shown in Fig. 6F. The example input (digit “9”) and the visualization of this larger network, demonstrating the flow of intensity to the output classification channels, are shown in Fig. 6 (G and H, respectively).

## DISCUSSION

We have realized an ultracompact and multifunctional photonic platform based on inverse design. An inverse designed 50:50 fixed coupler is realized, whose measured visibility and insertion loss are 0.996 and −0.48 dB, respectively. The photonic platform is composed of 86 IDFCs and 91 PSs, and the footprint is only 3 mm by 0.2 mm. We have experimentally verified the feasibility of our platform by performing quantum state evolution tasks with high fidelity and PNN with on-chip training. To further show the scalability of our proposed platform, simulation for 2D disorder SSH model and classification without PCA on the full digits dataset are theoretically realized. These results demonstrate the practicality of large scale inverse designed photonic platforms. The calculation power of our platform increases about one-order compared to other MZIs based platforms. This work provides crucial components for building large-scale ultracompact integrated photonic circuits and offers an integrated photonic platform for quantum simulations, optical neural networks, and optical information processing.

## MATERIALS AND METHODS

### Inverse design algorithm

We realize a self-developed software to inverse design the devices. We define an objective function as *f* = *f*(*E*(ε)) to represent the device’s performance, whose independent variable is *E*(ε). Here, *E* represents electric field and is the function of permittivity distribution ε. We use gradient descent algorithm to optimize ε.

$$\varepsilon_{i+1}=\varepsilon_{i}-\alpha \frac{\partial f}{\partial \varepsilon_{i}}$$ (15)

where *i* is the iteration times and α is the step length. It can be proved that

$$\frac{\partial f}{\partial \varepsilon_{i}}=-\omega^{2}\mu_{0}E\prime E$$ (16)

where ω is the circular frequency and *E*′ is the adjoint electric field, defined as

$$E\prime ={\left(\nabla \times \nabla \times -\omega^{2}\mu_{0}\varepsilon_{i}\right)}^{-1}\left(\frac{\partial f}{\partial E}\right)$$ (17)

We further use geometric restriction algorithm to optimize the device’s geometry shape and fitting the complementary metal-oxide semiconductor (CMOS) process limitation. We set a critical radius, which depends on the CMOS process, and we set it to be 240 nm. For islands or holes on the device whose size is less than half the critical radius, we use corrosion algorithms to shrink them until they disappear. For islands or holes on the device whose size is greater than half of the critical radius but still less than the critical radius, we use an expansion algorithm to expand them until they meet the requirements. The adjoint gradient algorithm and geometric restriction algorithm are applied simultaneously in the optimization process, so that the device can have good geometry while the performance is improved. Thus, the fabrication error can be reduced to make the devices have higher accuracy to improve the experimental performance.

### Fabrication

The devices were fabricated in CMOS pilot process line. The 248-nm λ deep ultraviolet (DUV) photolithography was used for the mass production. The device was fabricated on a 200-mm-diameter silicon-on-insulator wafer with a 220-nm-thick top silicon and a 3-μm-thick buried oxide layer. The wafer was coated with a positive photoresist and patterned by DUV lithography, then followed by double inductively coupled plasma etching process of top silicon. The waveguides of the device were formed by the 220-nm-depth full etch, and then the grating couplers were formed by the 70-nm-depth shallow etch. A high-temperature annealing process was adopted for reducing the optical loss of the waveguides. Then, a 1-μm-thick cladding silicon dioxide was deposited by plasma-enhanced chemical vapor deposition (PECVD) process before the heater and metallization process. A 55-nm-thick TiN layer was deposited by PECVD process and dry-etched to form the heater for the resistive thermal-optic PSs. Then, 800-nm-thick AlCu layer was deposited and wet-etched for the metallization pattern. Last, a 1-μm-thick cladding silicon dioxide was deposited by PECVD process and followed by the pad opening etching process to form the wire bonding pad.

### Packaging processes

The packaging processes were as follows:

1) Chip cleaning: Immerse the chip in a solution of configuration cleaning agent for ultrasonic cleaning and then dry it with nitrogen.

2) Mounting: Secure the chip onto the metal base, followed by curing it in an oven.

3) Wire bonding: Connect the chip to the PCB pads following the designed line sequence.

4) Optical alignment: Align the optical array with the chip’s waveguide port using a 6D adjustment frame for precise alignment. Monitor the optical output using a power meter until the loss parameter reaches its optimal value. Apply an appropriate amount of matching liquid to the contact end, scan the spectrum, and record the data after ultraviolet curing.

5) Heat curing and aging: Place the entire product in an oven for heat curing and aging. Remove the product upon completion.

6) Visual inspection: Inspect the product’s appearance, checking for any optical fiber damage, breakage, completeness, and correctness of the fiber connector channel identifier, stability of the optical fiber protection, and the integrity of the chip’s gold wires.

7) Packaging and warehousing: Package the product in designated packaging boxes to ensure it is protected during transportation, avoiding damage, shaking, or optical fiber breakage. After packaging is complete, affix a label with key information including the product name, specifications, and quantity.

### Experimental details

The laser source’s type is Santec TSL-510. Continuous-wave laser light (1550 nm, 20 mW) is equally divided into two parts, one is launched into the input port, and the other is launched into calibrated grating coupler. We use Thorlabs PM100USB and S154C to probe the power of output light. We use Cumec 512 channel dc power module MG-512-E-B-V1 to control the voltages of PSs.

We use a 10-channel fiber array to measure the output signal, corresponds to 10 grating couplers at the right side. The 1 and 10 grating couplers are connected by waveguide to calibrate the position of fiber array. The two to nine ports on fiber array are automatic aligned to the one to eight ports on platform, correspondingly. The input fiber is aligned to the grating coupler at the left side to input the signal. In the middle part of platform, there are eight groups of input and output grating coupler for calibration use.

## Supplementary Materials

### This PDF file includes:

Notes S1 to S10 Figs. S1 to S6 Tables S1 and S2 References
