# Supplementary material for: Ultracompact and multifunctional integrated photonic platform
Source: Sci Adv. 2024 Jun 19;10(25):eadm7569. doi: 10.1126/sciadv.adm7569 (PMC11186496; doi:10.1126/sciadv.adm7569)
Supplement: Supplementary file 1 — Notes S1 to S10 Figs. S1 to S6 Tables S1 and S2 References [file sciadv.adm7569_sm.pdf]

Science Advances
AAAS

Supplementary Materials for

Ultracompact and multifunctional integrated photonic platform

Zhuochen Du *et al.*

Corresponding author: Xiaoyong Hu, xiaoyonghu@pku.edu.cn; Cuicui Lu, cuicuilu@bit.edu.cn; Yan Yang, yyang10@ime.ac.cn

Sci. Adv. **10**, eadm7569 (2024)
DOI: 10.1126/sciadv.adm7569

This PDF file includes:

Notes S1 to S10
Figs. S1 to S6
Tables S1 and S2
References

Supplementary Note 1: Transfer matrix for network

The electric field in waveguide can be written as superposition of waveguide's eigenmode:

$$E(x,y,z)=\sum_m a_m(z)e^{i\beta_m z}E_m(x,y) \tag{S1}$$

Here we assume the waveguide is on z-direction. a_m, β_m and E_m are the amplitude, propagation constant and eigenmode, correspondingly. We only consider fundamental TE mode and we have two parallel waveguides, we use a ket-vector to represent the state in the two-waveguide system:

$$|\psi(z)\rangle=[a(z),b(z)]^T \tag{S2}$$

The function of inverse designed device can be represented by an operator, and its matrix from is:

$$\hat{F}=\begin{bmatrix}F_{11} & F_{12}\\ F_{21} & F_{22}\end{bmatrix} \tag{S3}$$

Due to the law of conservation of energy, we must have $\|\hat{F}\|_2\le 1$. And the theoretical insertion lossless condition is $\hat{F}^\dagger\hat{F}=1$.

Here the transfer matrix of inversed designed fixed coupler can be represented as:

$$\hat{F}_{IDFC}=\frac{1}{\sqrt{2}}\begin{bmatrix}1 & 1\\ 1 & -1\end{bmatrix} \tag{S4}$$

It satisfies the energy conservation law and theoretically lossless. And the matrix is the same as Hadamard Gate in quantum computation.

The matrix for phase shifter can be written as:

$$\hat{F}_{PS}=\begin{bmatrix}e^{i\phi} & 0\\ 0 & 1\end{bmatrix} \tag{S5}$$

We need 4 parameters to completely construct any $U(2)$ matrix. In our network we have two ways to construct an $U(2)$ matrix, shown in Fig.S1. Here we elaborate the first way. The transfer matrix is:

$$\hat{F}=\frac{1}{2}\begin{bmatrix}e^{i\phi_1} & 0\\ 0 & e^{i\phi_2}\end{bmatrix}\begin{bmatrix}1 & 1\\ 1 & -1\end{bmatrix}\begin{bmatrix}e^{i\phi_3} & 0\\ 0 & 1\end{bmatrix}\begin{bmatrix}1 & 1\\ 1 & -1\end{bmatrix}\begin{bmatrix}e^{i\phi_4} & 0\\ 0 & 1\end{bmatrix}$$

$$= \begin{bmatrix} e^{i\phi_1+\frac{i\phi_3}{2}+i\phi_4}\cos\frac{\phi_3}{2} & ie^{i\phi_1+\frac{i\phi_3}{2}}\sin\frac{\phi_3}{2} \\ ie^{i\phi_2+\frac{i\phi_3}{2}+i\phi_4}\sin\frac{\phi_3}{2} & e^{i\phi_2+\frac{i\phi_3}{2}}\cos\frac{\phi_3}{2} \end{bmatrix}$$

$$= e^{i\frac{\phi_1+\phi_2+\phi_3+\phi_4}{2}} \begin{bmatrix} e^{i\frac{\phi_1-\phi_2+\phi_4}{2}}\cos\frac{\phi_3}{2} & ie^{i\frac{\phi_1-\phi_2-\phi_4}{2}}\sin\frac{\phi_3}{2} \\ ie^{i\frac{-\phi_1+\phi_2+\phi_4}{2}}\sin\frac{\phi_3}{2} & e^{i\frac{-\phi_1+\phi_2-\phi_4}{2}}\cos\frac{\phi_3}{2} \end{bmatrix} \quad (S6)$$

To meet the general expression of an $U(2)$ matrix:

$$U(\alpha,\beta,\gamma,\delta) = e^{i\delta}\begin{bmatrix} e^{i\beta}\cos\alpha & e^{-i\gamma}\sin\alpha \\ -e^{i\gamma}\sin\alpha & e^{-i\beta}\cos\alpha \end{bmatrix} \quad (S7)$$

We have:

$$\begin{cases} \phi_3 = 2\alpha \\ \phi_1 - \phi_2 + \phi_4 = 2\beta \\ -\phi_1 + \phi_2 + \phi_4 = 2\gamma + \pi \\ \phi_1 + \phi_2 + \phi_3 + \phi_4 = 2\delta \end{cases} \quad (S8)$$

The solution for these equations is:

$$\begin{cases} \phi_1 = -\alpha + \beta + \delta \\ \phi_2 = -\alpha + \gamma + \delta + \frac{\pi}{2} \\ \phi_3 = 2\alpha \\ \phi_4 = \beta + \gamma + \frac{\pi}{2} \end{cases} \quad (S9)$$

Redo the same process and we can have the solution for the second way:

$$\begin{cases} \phi_1 = -\beta - \gamma - \frac{\pi}{2} \\ \phi_2 = 2\alpha \\ \phi_3 = -\alpha + \beta + \delta \\ \phi_4 = -\alpha + \gamma + \delta + \frac{\pi}{2} \end{cases} \quad (S10)$$

Supplementary Note 2: Method of generating input state and measuring complex output state

The initial state can be fully prepared, as illustrated in Figure S1(A). The phase modulators on the Mach-Zehnder interferometer (MZI) enable the generation of output with arbitrary beam splitter ratios. The arbitrary phase of the initial state can be achieved through the phase shifter in the last column.

The complex output part of the computing platform can be used to measure the phase of

output state if desired. We can use the principal of interference to determine the phase difference between the two nearest waveguides. Here we take the first two waveguides as an example. As shown in Fig.S1 (B), we control the number 79 PSs and measure the output power of out port 1 and 2. Meanwhile, PSs from number 80 to 91 remain identity. The reduced output state is:

$$\frac{1}{2}\begin{bmatrix}1 & 1\\ 1 & -1\end{bmatrix}\begin{bmatrix}e^{i\phi_{79}} & 0\\ 0 & 1\end{bmatrix}\begin{bmatrix}1 & 1\\ 1 & -1\end{bmatrix}\begin{bmatrix}a_1 e^{i\theta_1}\\ a_2 e^{i\theta_2}\end{bmatrix} = e^{\frac{i\phi_{79}}{2}}\begin{bmatrix}a_1 e^{i\theta_1}\cos\frac{\phi_{79}}{2} + ia_2 e^{i\theta_2}\sin\frac{\phi_{79}}{2}\\ ia_1 e^{i\theta_1}\sin\frac{\phi_{79}}{2} + a_2 e^{i\theta_2}\cos\frac{\phi_{79}}{2}\end{bmatrix} \qquad (S11)$$

The output power for the first port reaches its max when:

$$\tan\phi_{79} = \frac{2a_1 a_2 \sin(\theta_1 - \theta_2)}{a_1^2 - a_2^2} \qquad (S12)$$

Once we confirm this specific value of ϕ_{79}, we can calculate the value of $\theta_1 - \theta_2$. In fact, we could simultaneously obtain each $\theta_{2k-1} - \theta_{2k}$ for $k = 1,2,3,4$. Second step, we control the number 88-90 PSs to calculate $\theta_{2k} - \theta_{2k+1}$ for $k = 2,3,4$. In this way, we could confirm all the phase of final state up to a common trivial phase factor.

Supplementary Note 3: Fitting parameters for transmission versus voltage curve

We measured the transmission versus bias using test structure shown in the main text. We fit the transmission as $T = a + b\cos(cV^2 + d)$. The initial guess is $a = b = 0.5, c = 0.1, d = 0$ for same side and $d = \pi$ for opposite side. The measured 2π voltage is 7.4V. The results shown in Table.S1.

Supplementary Note 4: On-chip calibration by gradient descent algorithm

The slight error of device will accumulate a non-negligible error and influence the calculating result. So, the calibration for the chip is prerequisite. We construct a function relationship between the calibrated phase shifting and the initial desired phase shifting:

$$\phi_i^{(r)} = f_i(v_i) \qquad (S13)$$

Here, $\phi_i^{(r)}$ represent the calibrated phase shifting, v_i is the voltage added on the ith PS and f_i is the function. The form of the function is uncertain and our method is to use a quadratic function to approximate represent the relationship:

$$\phi_i^{(r)} = a_i + b_i v_i + c_i v_i^2 \qquad (S14)$$

We add the linear term here to more accurately describe the model. The parameter a_i, b_i, c_i are to be optimized. We define the loss function as:

$$LOSS = \sum_{i=1}^{N}\left[1 - \left(\sum_{j=1}^{8}\sqrt{p_j q_j}\right)\right] \tag{S15}$$

$\sum_{j=1}^{8}\sqrt{p_j q_j}$ is the fidelity of the measuring result, p_j and q_j are the measured and theoretical normalized intensity distribution of the jth port, correspondingly. Here we measure the $0, \frac{\pi}{2}, \pi$ and 2π-voltage of every phase shifter to fit the parameters.

We measure 1000 random unitary matrix to test the calibration result. The fidelity frequency distribution is shown in Fig.S2(a). The averaged measure fidelity is 99.20 (53) %. This demonstrates the effective of our calibration. We further test the robustness of our platform. We add a normal distribution perturbation on the phase shifters. We measured 100 sets of data for every standard deviation, who changes from 0 to 2. The results show the platform has strong robustness when standard deviation below 0.4. Even standard deviation reaches 0.6, the fidelity is still above 0.6. This result guarantees the accuracy of our measurements.

Supplementary Note 5: Trotter equations

Consider a Hamiltonian consist of two parts $H = H_1 + H_2$ and $[H_1, H_2] \neq 0$. The evolution operator $U = \exp(-iHt) \neq \exp(-iH_1 t)\exp(-iH_2 t)$. An approximation method to divide the total time into many small parts, i.e.

$$U \approx \prod_{i=1}^{N} \exp(-iH_1\Delta t)\exp(-iH_2\Delta t) \tag{S16}$$

Here $\Delta t = \frac{t}{N}$, the error is $O[(\Delta t)^2]$. This is the first order Trotter approximation. A more precise method is to use second order Trotter approximation:

$$U \approx \prod_{i=1}^{N} \exp\left(-\frac{iH_1\Delta t}{2}\right)\exp(-iH_2\Delta t)\exp\left(-\frac{iH_1\Delta t}{2}\right) \tag{S17}$$

It can be proved that the error reduces to $O[(\Delta t)^3]$ in this case. Here we compare the fidelity of First and Second Order Trotter for 1D SSH model equation of the function of N. The results for both trivial and topological phase are shown in Fig.S3. It clear that the second order method has higher fidelity and the accuracy increase with N.

Supplementary Note 6: Additional results for Floquet SSH model

Fig.S4(a)-(b) shows the calculated period boundary condition quasienergy band at the phase transition point. The $(u,v)=(\pi/4,3\pi/4)$ means $u=\pi/4, v=3\pi/4$ or $u=3\pi/4, v=\pi/4$. When the parameter changes along the path from A to B, the energy gap closes at 0 and then reopen. An edge state emerges at 0 energy, indicating a topological phase transition. This transition is similar with the normal SSH model. The parameters keep moving from B to C, the gap at π closes and reopen. An edge state emerges at π energy, indicating a topological phase transition. In this case, the system has both 0 modes and π modes edge state. From C to D, the energy gap closes at 0 and then reopen. The 0 modes edge state vanish, only remain π modes. Finally, from D to A, the energy gap closes at π and then reopen, no edge state exists, the system is back to trivial condition. Fig.S4(c)-(d) show the quasienergy band for $(u,v)=(\pi/4,\pi/8)$ and $(u,v)=(3\pi/8,\pi/4)$.

The calculated edge states are shown in Fig.S4(e)-(f). Fig.S4(e) shows the 0-modes for $u=\pi/4, v=\pi/2$ and $u=\pi/2, v=3\pi/4$. Fig.S4(f) shows the π-modes for $u=3\pi/4, v=\pi/2$ and $u=\pi/2, v=3\pi/4$. The blue and orange line represent the real and imaginary part correspondingly.

Four sets of parameters of Floquet SSH model are also simulated: 1) $ut_1=\frac{\pi}{8}, vt_2=\frac{\pi}{4}$, 2) $ut_1=\frac{\pi}{4}, vt_2=\frac{\pi}{8}$, 3) $ut_1=\frac{\pi}{4}, vt_2=\frac{3\pi}{8}$, 4) $ut_1=\frac{3\pi}{8}, vt_2=\frac{\pi}{4}$. The simulation results are shown in Fig. S4(g)-(j).

Supplementary Note 7: Dataset and preprocessing

We downloaded the dataset from the following website, https://archive.ics.uci.edu/dataset/80/optical+recognition+of+handwritten+digits, which uses the preprocessing programs made available by NIST to extract normalized bitmaps of handwritten digits from a preprinted form. The data was generated from a total of 43 people, 30 contributed to the training set and different 13 to the test set. 32x32 bitmaps are divided into nonoverlapping blocks of 4x4 and the number of on pixels are counted in each block to form an input matrix of 8x8 where each element is an integer in the range 0...16. This reduces dimensionality and gives invariance to small distortions, with a total of 3823 training images

and 1797 testing images. We first rescaled the values to 0 to 1, and uses Principle Component Analysis (PCA) supported by the sklearn library for preprocessing. We keep only the first 8 components of the dataset, and effectively reduces the dimensions of the images to 8. The vectors are then gathered and selected based on their labels to form the training and testing dataset of the 4-class and 8-class problems. The input is encoded so that the intensity is the absolute value, and a π phase is added to negative values.

Supplementary Note 8: Additional results for 2D SSH model

Here we theoretically perform a simulation for 2D SSH model without disorder. We use second order Trotter equation and set $u = 1, v = 2$ or $u = 2, v = 1$. The initial state populate at both corner and bulk are considered. The results are shown in Fig.S5. Fig.S5(a) shows the results for $u = 2, v = 1, |\psi(0)\rangle = |1\rangle|1\rangle$. We set $t = 0,1,2,3,4,5$ to show the evolution. Fig.S5(b) shows the results for $u = 1, v = 2, |\psi(0)\rangle = |1\rangle|1\rangle$. Fig.S4(c) shows the results for $u = 2, v = 1, |\psi(0)\rangle = |16\rangle|16\rangle$. Fig.S5(d) shows the results for $u = 1, v = 2, |\psi(0)\rangle = |16\rangle|16\rangle$.

In addition, we calculate the topological phase diagram for disorder 2D SSH model with $\sigma_u = \sigma_v = \sigma$. The result is shown in Fig.S6(a). We choose $u_0 = 0.5, \sigma = 0.5$, $u_0 = 1.2, \sigma = 0.2$ and $u_0 = 1.2, \sigma = 1$ to simulate the evolution of AMCD. The results are shown in Fig.S6(b)-(d), correspondingly. Topological phase diagram for $\sigma_v = 2\sigma_u = 2\sigma$ is shown in Fig.S6(e). Simulations for evolution of AMCD are shown in Fig.S6(f)-(h).

Supplementary Note 9: Comparison with other MZI based quantum simulations

Here we make a brief comparison between our work and other MZI based quantum simulation works. The results are illustrated in Table S2. From fidelity perspective, our work aligns with the current state of the art. This validates the reliability of the platform we have proposed.

Supplementary Note 10: Method for measuring quasienergy band

We propose a scheme to realize the measurement of quasienergy mediately. In the Floquet SSH model, due to the presence of spatial translational symmetry, quasi-momentum is a good quantum number that commutes with the Hamiltonian. Therefore, quasimomentum can be employed to characterize the energy bands. Here, we specify a quasimomentum to measure the

pseudo-energy.

$$U(k_x) = \exp(-iH_2 t_2)\exp(-iH_1 t_1) \tag{S18}$$

$$H_1 = \begin{bmatrix} 0 & u \\ u & 0 \end{bmatrix} \qquad \exp(-iH_1 t_1) = \begin{bmatrix} \cos ut_1 & -i\sin ut_1 \\ -i\sin ut_1 & \cos ut_1 \end{bmatrix}$$

$$H_2 = \begin{bmatrix} 0 & ve^{-ik_x} \\ ve^{ik_x} & 0 \end{bmatrix} \qquad \exp(-iH_2 t_2)$$

$$= \begin{bmatrix} \cos vt_2 & -ie^{-ik_x}\sin vt_2 \\ -ie^{ik_x}\sin vt_2 & \cos vt_2 \end{bmatrix}$$

The eigenvalues and corresponding eigenstates of the evolution operator can be determined through the application of the gradient descent algorithm. Take the trial state as $|\psi(\theta,\phi)\rangle = [\cos\theta\,, \mathrm{e}^{i\phi}\sin\theta]^T$. The output state is $U|\psi\rangle$. Assuming the eigenvalues and eigenstates of U are $e^{-iE_{1,2}T}$ and $|1\rangle$, $|2\rangle$ respectively.

$$U|\psi\rangle = U(\alpha_1|1\rangle + \alpha_2|2\rangle) = \alpha_1 e^{-iE_1T}|1\rangle + \alpha_2 e^{-iE_2T}|2\rangle \tag{S19}$$

Here $\alpha_{1,2}$ are the expansion coefficients that satisfy $|\alpha_1|^2 + |\alpha_2|^2 = 1$. Define objective function as

$$F(\theta,\phi) = |\langle\psi|U|\psi\rangle|^2 = |\alpha_1|^4 + |\alpha_2|^4 + 2|\alpha_1|^2|\alpha_2|^2\cos(E_1 - E_2)T \tag{S20}$$

Take the notice of

$$F(\theta,\phi) \le |\alpha_1|^4 + |\alpha_2|^4 + 2|\alpha_1|^2|\alpha_2|^2 = 1 \tag{S21}$$

$F = 1$ if and only if $|\alpha_1| = 1$ or $|\alpha_2| = 1$. In this case, the trial state is the eigenstate of the evolution operator. After obtaining the eigenstates, measuring the phase difference between the output state resulting from the action of the evolution operator on these eigenstates and the light transmitted through the reference waveguide allows for the determination of the corresponding eigenvalues.

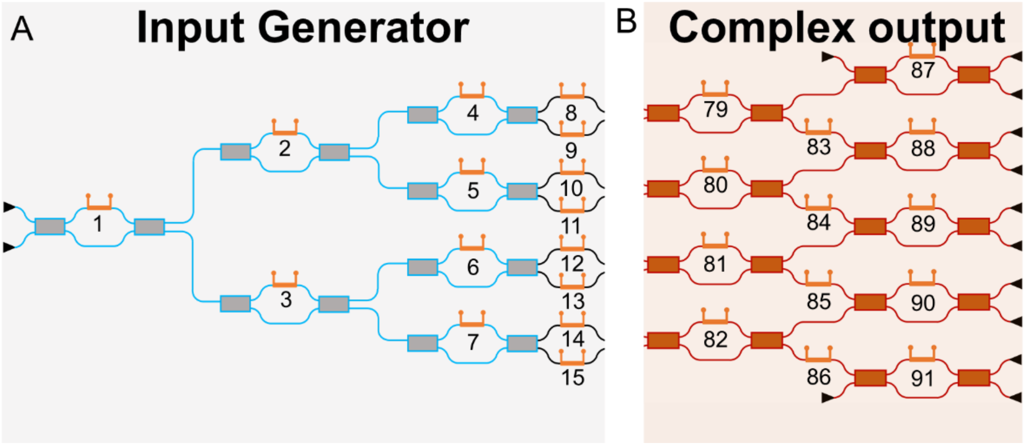

Figure S1 Scheme of input generator and complex output. (A) The input generator completely prepares the input states, with phase shifters 1-7 regulating amplitude and phase shifters 8-15 managing the phase. (B) The number of PSs is labelled in the figure, from 79-91. This section facilitates the measurement of the output phase.

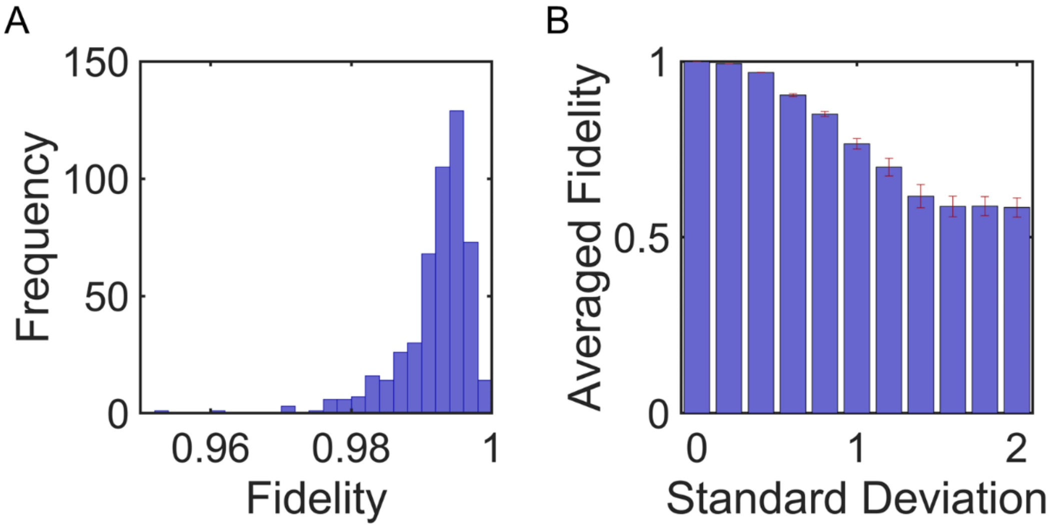

Figure S2 Test Results. (a) The frequency distribution for 1000 random unitary matrixes. These results confirm the effectiveness of calibration. (b) Robustness test result. Adding a normal distribution perturbation on voltage. The measured averaged fidelity decreases as the standard deviation increase. The fidelity is above 0.9 even the standard deviation is 0.6.

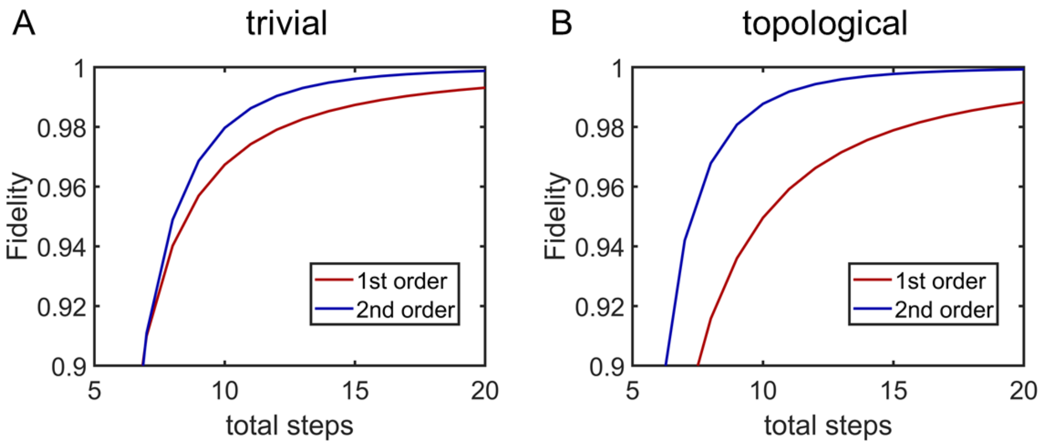

Figure S3 Calculated fidelity for Trotter equations. (a) Results for SSH trivial case, the parameters are $u = 2, v = 1$. The total simulation time is uniformly separated into several parts, as an independent variable. The dependent variable is the fidelity. Here the fidelity increased with total steps. The red line and blue line represent first order Trotter equation and second order Trotter equation, correspondingly. It's clear that the fidelity for second order is higher. (b) Results for SSH topological case, the parameters are $u = 1, v = 2$. The conclusion remains same as the trivial case. These results confirm the higher accuracy of second order Trotter equation.

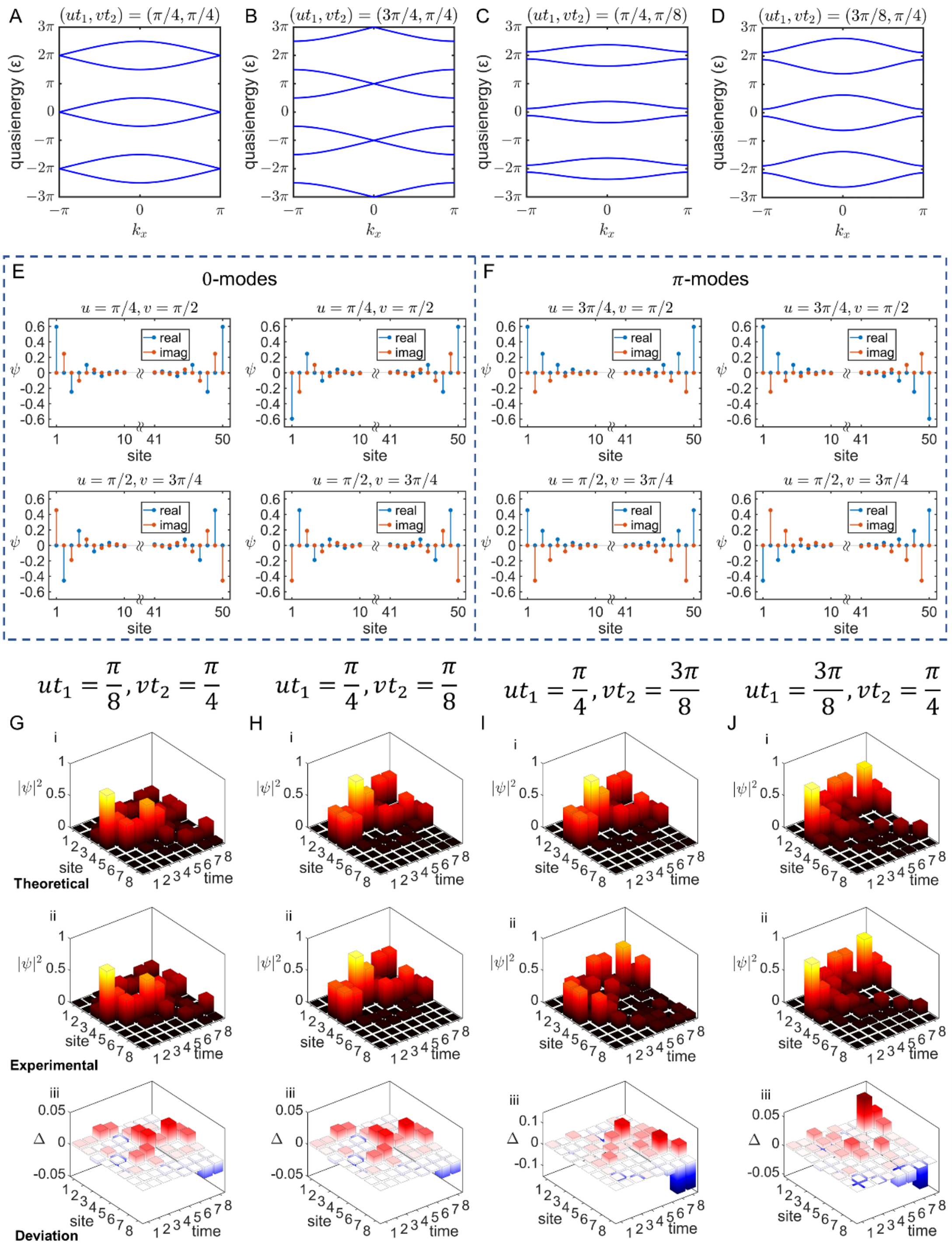

Figure S4 Additional results for Floquet evolved SSH. (a) Quasienergy band at topological phase transition point $(ut_1, vt_2) = (\pi/4, \pi/4)$. The gap closes at $k_x = \pi$ and $\varepsilon = 0$. (b) Quasienergy band at topological phase transition point $(ut_1, vt_2) = (\pi/4, 3\pi/4)$. The gap closes at $k_x = 0$ and $\varepsilon = \pi$. (c) Quasienergy band for $(ut_1, vt_2) = (\pi/4, \pi/8)$. (d) Quasienergy band for $(ut_1, vt_2) = (3\pi/8, \pi/4)$. (e) Calculated 0-mode edge state for $u = \pi/4, v = \pi/2$ and $u = \pi/2, v = 3\pi/4$. The blue and orange point represent the real and

imaginary part, correspondingly. (f) Calculated π-mode edge state for $u = 3\pi/4, v = \pi/2$ and $u = \pi/2, v = 3\pi/4$. The blue and orange point represent the real and imaginary part, correspondingly. (g) Quantum simulation results for $ut_1 = \pi/8, vt_2 = \pi/4$. Here i, ii and iii represent theoretical, experimental results and deviation, correspondingly. (h) Quantum simulation results for $ut_1 = \pi/4, vt_2 = \pi/8$. (i) Quantum simulation results for $ut_1 = \pi/4, vt_2 = 3\pi/8$. (j) Quantum simulation results for $ut_1 = 3\pi/8, vt_2 = \pi/4$.

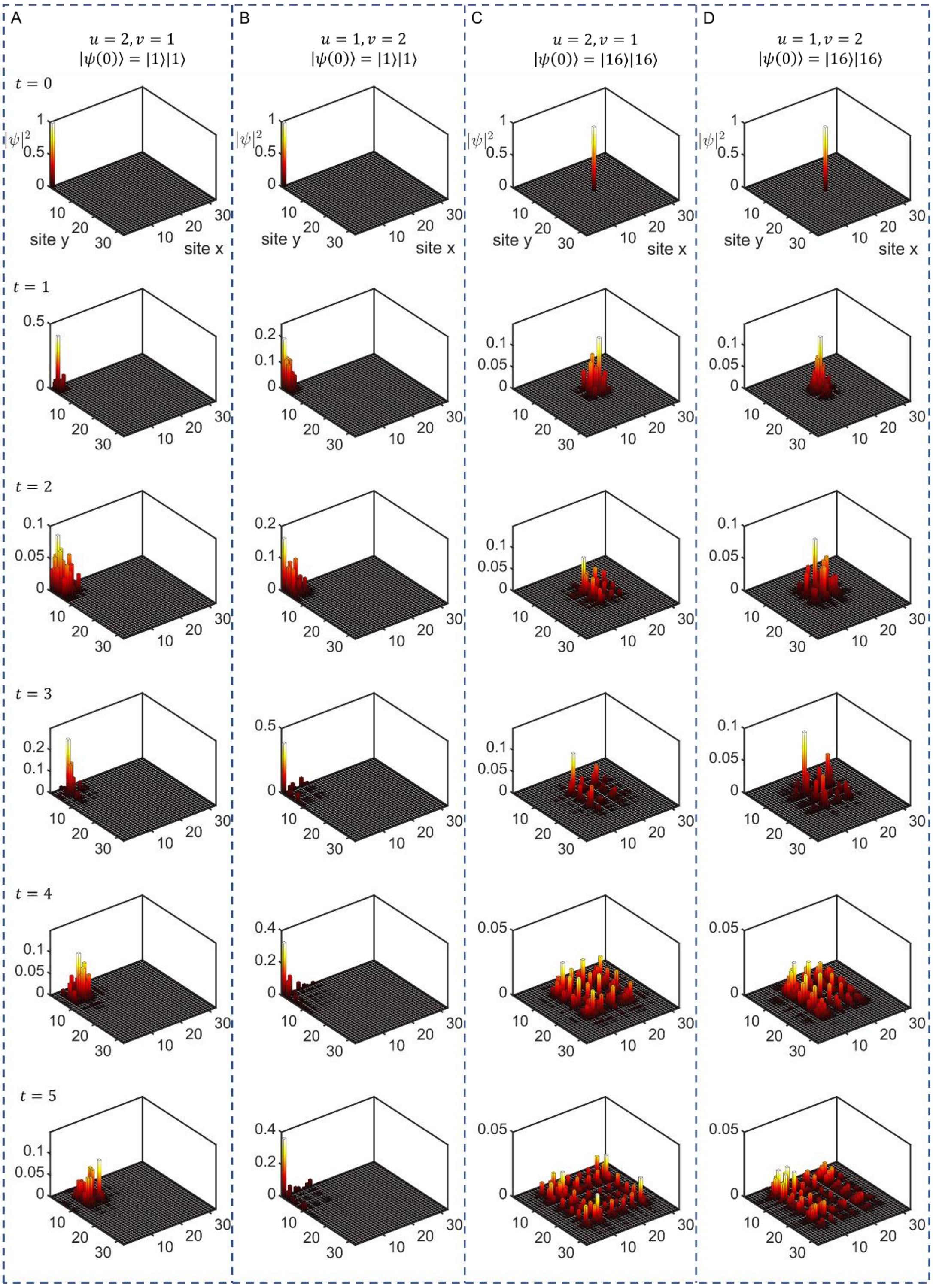

Figure S5 Additional results for 2D SSH model. (a) Simulation results for $u = 2, v = 1$. Here the x-axis and y-axis represent the site index on x direction and y direction, correspondingly. Total number of sites is 32. The initial state is chosen as $|\psi(0)\rangle = |1\rangle|1\rangle$. The evolution time is from 0 to 5 and time step is 1. Here we use second order Trotter equation to perform the simulation. The population spread into bulk with the time evolved, indicating the trivial property. (b) Simulation results for $u = 1, v = 2$. Population stays at corner due to the topology. (c)

Simulation results for $u = 2, v = 1$ with the initial state $|\psi(0)\rangle = |16\rangle|16\rangle$. (d) Simulation results for $u = 1, v = 2$ with the initial state $|\psi(0)\rangle = |16\rangle|16\rangle$.

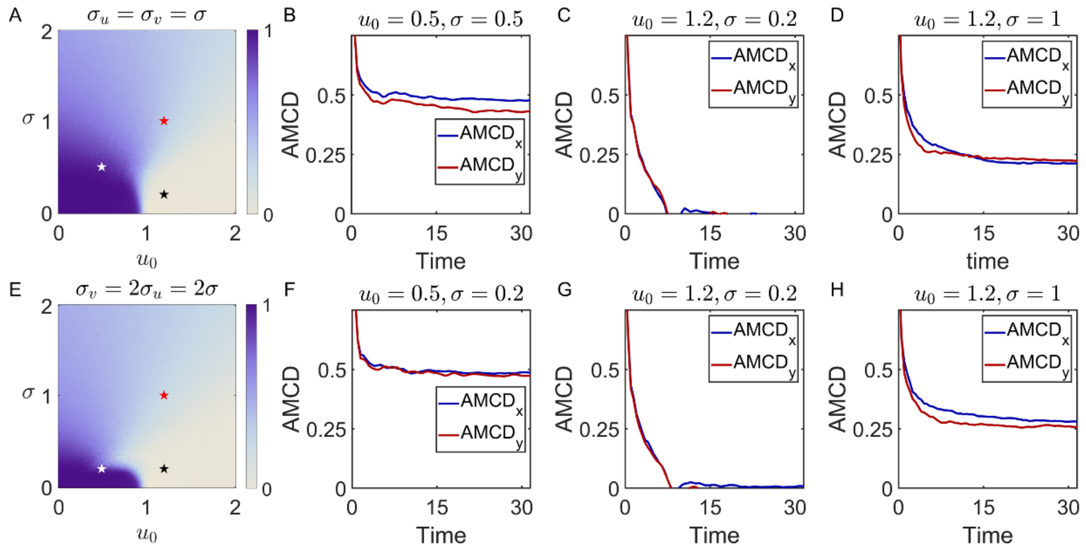

Figure S6 Additional results for disorder 2D SSH model. (a) Topological phase diagram for disorder 2D SSH model with $\sigma_u = \sigma_v = \sigma$. For simplicity, we plot 2×AMCD here which is the winding number. Three points labeled by white, red and black star are chosen to perform simulation and calculate the AMCD. The results are averaged after 1000 times calculation. (b) Calculated AMCD for $u_0 = 0.5, \sigma = 0.5$. (c) Calculated AMCD for $u_0 = 1.2, \sigma = 0.2$. (b) Calculated AMCD for $u_0 = 1.2, \sigma = 1$. (e) Topological phase diagram for disorder 2D SSH model with $\sigma_v = 2\sigma_u = 2\sigma$. (f) Calculated AMCD for $u_0 = 0.5, \sigma = 0.2$. (g) Calculated AMCD for $u_0 = 1.2, \sigma = 0.2$. (h) Calculated AMCD for $u_0 = 1.2, \sigma = 1$.

Table.S1 Fitting parameters for testing MZI

	a	b	c	d	r^2
T_{31}	0.492	0.481	0.113	-0.321	0.996
T_{41}	0.496	0.491	0.113	2.682	0.9993
T_{32}	0.500	0.498	0.113	2.752	0.9997
T_{42}	0.501	0.484	0.112	-0.479	0.998

Table.S2 Comparison of fidelity

	Model	Fidelity
**This work**	Floquet SSH	97.90%
**This work**	AAH	99.34%
**Harris et al [19]**	Disordered quantum walk	99.76-99.90%
**Wang et al [58]**	2D SSH	96.72-97.43%
**Wang et al [58]**	BBH	96.98-97.66%
**Konoike et al [59]**	Quantum walk	97-98%
**Mower et al [60]**	Quantum random walk	99.77%

REFERENCES AND NOTES

1. R. Salem, M.A. Foster, A.L. Gaeta. Application of space–time duality to ultrahigh-speed optical signal processing. *Adv. Opt. Photon.* **5**, 274–317 (2013).

2. S. Salahuddin, K. Ni, S. Datta. The era of hyper-scaling in electronics. *Nat. Electron.* **1**, 442–450 (2018).

3. P. P. Absil, P. Verheyen, P. De Heyn, M. Pantouvaki, G. Lepage, J. De Coster, J. Van Campenhout. Silicon photonics integrated circuits: A manufacturing platform for high density, low power optical I/O's. *Opt. Express* **23**, 9369–9378 (2015).

4. M. R. J. Azizpour, M. Soroosh, N. Dalvand, Y. Seifi-Kavian, All-optical ultra-fast graphene-photonic crystal switch. *Crystals* **9**, 461 (2019).

5. B. Zhen, C. W. Hsu, Y. Igarashi, L. Lu, I. Kaminer, A. Pick, S. Ll Chua, J. D. Joannopoulos, M. Soljačić, Spawning rings of exceptional points out of Dirac cones. *Nature* **525**, 354–358(2015).

6. Q. Xiao, C. Klitis, S. Li, Y. Chen, X. Cai, M. Sorel, S. Yu, Generation of photonic orbital angular momentum superposition states using vortex beam emitters with superimposed gratings. *Opt. Express* **24**, 3168–3176 (2016).

7. G. Zhao, T. Zhao, H. Xiao, Z. Liu, G. Liu, J. Yang, Z. Ren, J. Bai, Y. Tian, Tunable Fano resonances based on microring resonator with feedback coupled waveguide. *Opt. Express* **24**, 20187–20195 (2016).

8. T. Chen, M. Pauly, P. M. Reis, A reprogrammable mechanical metamaterial with stable memory. *Nature* **589**, 386–390 (2021).

9. A.Silva, F. Monticone, G. Castaldi, V. Galdi, A. Alù, N. Engheta. Performing mathematical operations with metamaterials. *Science* **343**, 160–163 (2014).

10. J. Lin, J. P. B. Mueller, Q. Wang, G. Yuan, N. Antoniou, X.-C. Yuan, F. Capasso, Polarization-controlled tunable directional coupling of surface plasmon polaritons. *Science* **340**, 331–334 (2013).

11. P. Nagpal, N. C. Lindquist, S.-H. Oh, D. J. Norris, Ultrasmooth patterned metals for plasmonics and metamaterials. *Science* **325**, 594–597 (2009).

12. A. Annoni, E. Guglielmi, M. Carminati, G. Ferrari, M. Sampietro, D. AB Miller, A. Melloni, F. Morichetti, Unscrambling light—automatically undoing strong mixing between modes. *Light Sci Appl.* **6**, e17110 (2017).

13. X. Xu, G. Ren, T. Feleppa, X. Liu, A. Boes, A. Mitchell, A. J. Lowery, Self-calibrating programmable photonic integrated circuits. *Nat. Photonics* **16**, 595–602 (2022).

14. H. Zhou, Y. Zhao, X. Wang, G. Dingshan, J. Dong, X. Zhang, Self-configuring and reconfigurable silicon photonic signal processor. *ACS Photonics* **7**, 792–799 (2020).

15. J. B. Spring, B. J. Metcalf, P. C. Humphreys, W. S. Kolthammer, X.-M. Jin, M. Barbieri, A. Datta, N. Thomas-Peter, N. K. Langford, D. Kundys, J. C. Gates, B. J. Smith, P. G. R. Smith, I. A. Walmsley, Boson sampling on a photonic chip. *Science* **339,** 798–801 (2013).

16. M. A. Broome, A. Fedrizzi, S. Rahimi-Keshari, J. Dove, S. Aaronson, T. C. Ralph, A. G. White, Photonic boson sampling in a tunable circuit. *Science* **339**, 794–798 (2013).

17. A. Crespi, R. Osellame, R. Ramponi, D. J. Brod, E. F. Galvão, N. Spagnolo, C. Vitelli, E. Maiorino, P. Mataloni F. Sciarrino, Integrated multimode interferometers with arbitrary designs for photonic boson sampling. *Nat. Photonics* **7**, 545–549 (2013).

18. M. Tillmann, B. Dakić, R. Heilmann, S. Nolte, A. Szameit, P. Walther, Experimental boson sampling. *Nat. Photonics* **7**, 540–544 (2013).

19. N. C. Harris, G. R. Steinbrecher, M. Prabhu, Y. Lahini, J. Mower, D. Bunandar, C. Chen, F. N. C. Wong, T. Baehr-Jones, M. Hochberg, S. Lloyd, D. Englund, Quantum transport simulations in a programmable nanophotonic processor. *Nat. Photonics* **11**, 447–452 (2017).

20. A. Crespi, R. Osellame, R. Ramponi, V. Giovannetti, R. Fazio, L. Sansoni, F. De Nicola, F. Sciarrino, P. Mataloni, Anderson localization of entangled photons in an integrated quantum walk. *Nat. Photonics* **7**, 322–328 (2013).

21. A. Peruzzo, M. Lobino, J. C. F. Matthews, N. Matsuda, A. Politi, K. Poulios, X.-Q. Zhou, Y. Lahini, N. Ismail, K. Wörhoff, Y. Bromberg, Y. Silberberg, M. G. Thompson, J. L. O'Brien, Quantum Walks of Correlated Photons. *Science* **329**, 1500–1503(2010)

22. L. Sansoni, F. Sciarrino, G. Vallone, P. Mataloni, A. Crespi, R. Ramponi, R. Osellame, Two-particle bosonic-fermionic quantum walk via integrated photonics. *Phys. Rev. Lett.* **108**, 010502 (2012).

23. M. Ogrodnik, S. Miwa, T. Tchkonia, D. Tiniakos, C. L. Wilson, A. Lahat, C. P. Day, A. Burt, A. Palmer, Q. M. Anstee, S. N. Grellscheid, J. H. J. Hoeijmakers, S. Barnhoorn, D. A. Mann, T. G. Bird, W. P. Vermeij, J. L. Kirkland, J. F. Passos, T. von Zglinicki, D. Jurk, Cellular senescence drives age-dependent hepatic steatosis. *Nat. Commun.* **8**, 15691 (2017).

24. A. Peruzzo, J. McClean, P. Shadbolt, M. Yung, X. Zhou, P. Love, A. Aspuru-Guzik, J. O'Brien, A variational eigenvalue solver on a photonic quantum processor. *Nat. Commun.* **5**, 4213 (2014).

25. J. Huh, G. G. Guerreschi, B. Peropadre, J. R. McClean, A. Aspuru-Guzik, Boson sampling for molecular vibronic spectra. *Nat. Photonics* **9**, 615–620 (2015).

26. J. Wang, S. Paesani, R. Santagati, S. Knauer, A. Gentile, N. Wiebe, M. Petruzzella, J. O'Brien, J. Rarity, A. Laing, M. Thompson, Experimental quantum Hamiltonian learning. *Nat. Phys.* **13**, 551–555 (2017).

27. C. Sparrow, E. Martín-López, N. Maraviglia, A. Neville, C. Harrold, J. Carolan, Y. N. Joglekar, T. Hashimoto, N. Matsuda, J. L. O’Brien, D. P. Tew, A. Laing, Simulating the vibrational quantum dynamics of molecules using photonics. *Nature* **557**, 660–667 (2018).

28. X. Qiang, X. Zhou, J. Wang, C. M. Wilkes, T. Loke, S. O’Gara, L. Kling, G. D. Marshall, R. Santagati, T. C. Ralph, J. B. Wang, J. L. O’Brien, M. G. Thompson, J. C. F. Matthews, Large-scale silicon quantum photonics implementing arbitrary two-qubit processing. *Nat. Photonics* **12**, 534–539 (2018).

29. J. Carolan, C. Harrold, C. Sparrow, E. Martín-López, N. J. Russell, J. W. Silverstone, P. J. Shadbolt, N. Matsuda, M. Oguma, M. Itoh, G. D. Marshall, M. G. Thompson, J. C. F. Matthews, T. Hashimoto, J. L. O’Brien, A. Laing, Universal linear optics. *Science* **349**, 711–716(2015).

30. A. Politi, M. J. Cryan, J. G. Rarity, S. Yu, J. L. O'Brein, Silica-on-silicon waveguide quantum circuits. *Science* **320**, 646–649 (2008).

31. A. Politi, J. C. F. Matthews, J. L. O'Brein, Shor's Quantum Factoring Algorithm on a Photonic Chip. *Science* **325**, 1221–1221(2009).

32. D. Llewellyn, Y. Ding, I. I. Faruque, S. Paesani, D. Bacco, R. Santagati, Y.-J. Qian, Y. Li, Y.-F. Xiao, M. Huber, M. Malik, G. F. Sinclair, X. Zhou, K. Rottwitt, J. L. O'Brien, J. G. Rarity, Q. Gong, L. K. Oxenlowe, J. Wang, M. G. Thompson, Chip-to-chip quantum teleportation and multi-photon entanglement in silicon. *Nat. Phys.* **16**, 148–153 (2020).

33. F. Flamini, L. Magrini, A. S. Rab, N. Spagnolo, V. D'Ambrosio, P. Mataloni, F. Sciarrino, T. Zandrini, A. Crespi, R. Ramponi, R. Osellame, Thermally reconfigurable quantum photonic circuits at telecom wavelength by femtosecond laser micromachining. *Light Sci. Appl.* **4**, e354 (2015).

34. J. Wang, S. Paesani, Y. Ding, R. Santagati, P. Skrzypczyk, A. Salavrakos, J Tura, R. Augusiak, L. Mančinska, D. Bacco, D. Bonneau, J. W. Silverstone, Q. Gong, A. Acín, K. Rottwitt, L. K. Oxenløwe, J. L. O'Brien, A. Laing, M. G. Thompson, Multidimensional quantum entanglement with large-scale integrated optics. *Science* **360**, 285–291 (2018).

35. J. W. Silverstone, R. Santagati, D. Bonneau, M. J. Strain, M. Sorel, J. L. O'Brien, M. G. Thompson, Qubit entanglement between ring-resonator photon-pair sources on a silicon chip. *Nat. Commun.* **6**, 7948 (2015).

36. Y. Shen, N. C. Harris, S. Skirlo, M. Prabhu, T. Baehr-Jones, M. Hochberg, X. Sun, S. Zhao, H. Larochelle, D. Englund, M. Soljačić, M. Deep learning with coherent nanophotonic circuits. *Nat. Photon.* **11**, 441–446 (2017).

37. T. W. Hughes, M. Minkov, Y. Shi, S. Fan, Training of photonic neural networks through in situ backpropagation and gradient measurement. *Optica* **5**, 864–871 (2018).

38. G. R. Steinbrecher, J. P. Olson, D. Englund, J. Carolan, Quantum optical neural networks. *npj Quantum Inf.* **5**, 60 (2019).

39. K. Liao, C. Li, T. Dai, C. Zhong, H. Lin, X. Hu, Q. Gong, Matrix eigenvalue solver based on reconfigurable photonic neural network. *Nanophotonics* **11**, 4089–4099 (2022).

40. K. Liao, Y. Chen, Z. Yu, X. Hu, X. Wang, C. Lu. H. Lin, Q. Du, J. Hu, Q. Gong, All-optical computing based on convolutional neural networks. *Opto-Electron Adv.* **4**, 200060 (2021).

41. R. P. Feynman, *Simulating physics with computers* (CRC Press, 2018), pp. 133–153.

42. M. A. Nielsen, I. L. Chuang, *Quantum computation and quantum information* (Cambridge Univ. Press, 2010).

43. A. J. Heeger, S. Kivelson, J. R. Schrieffer, W. -P. Su, Solitons in conducting polymers. *Rev. Mod. Phys.* **60**, 781–850 (1988).

44. M. C. Rechtsman, J. M. Zeuner, Y. Plotnik, Y. Lumer, D. Podolsky, F. Dreisow, S. Nolte, M. Segev, A. Szameit, Photonic Floquet topological insulators. *Nature* **496**, 196–200 (2013).

45. W. A. Benalcazar, B. A. Bernevig, T. L. Hughes, Quantized electric multipole insulators. *Science* **357**, 61–66 (2017).

46. Y. E. Kraus, Y. Lahini, Z. Ringel, M. Verbin, O. Zilberberg. Topological states and adiabatic pumping in quasicrystals. *Phys. Rev. Lett.* **109**, 106402 (2012).

47. S. Aubry, G. André. Analyticity breaking and Anderson localization in incommensurate lattices. *Ann. Israel Phys. Soc.* **3**, 133 (1980).

48. P. G. Harper, Single band motion of conduction electrons in a uniform magnetic field. *Proc. Phys. Soc. A* **68**, 874–878 (1955).

49. F. Liu, S. Ghosh, Y. D. Chong, Localization and adiabatic pumping in a generalized Aubry-André-Harper model. *Phys. Rev. B* **91**, 014108 (2015).

50. X. Lin, Y. Rivenson, N. T. Yardimci, M. Veli, Y. Luo, M. Jarrahi, A. Ozcan, All-optical machine learning using diffractive deep neural networks. *Science* **361**,1004–1008(2018).

51. Z.Y. Duan, H. Chen and X. Lin, Optical multi-task learning using multi-wavelength diffractive deep neural networks. *Nanophotonics* **12**, 893–903 (2023).

52. E. Alpaydin, C. Kaynak, *Optical Recognition of Handwritten Digits* (UCI Machine Learning Repository, 1998).

53. Y. LeCun, L. Bottou, Y. Bengio, P. Haffner, Gradient-based learning applied to document recognition. *Proc. IEEE* **86**, 2278–2324 (1998).

54. X. Shi, I. Kiorpelidis, R. Chaunsali, V. Achilleos, G. Theocharis, J. Yang, Disorder-induced topological phase transition in a one-dimensional mechanical system. *Phys. Rev. Res.* **3**, 033012 (2021).

55. Y. Wang, Y.-H. Lu, F. Mei, J. Gao, Z.-M. Li, H. Tang, S.-L. Zhu, S. Jia, X.-M. Jin, Direct observation of topology from single-photon dynamics. *Phys. Rev. Lett.* **122**, 193903 (2019).

56. D. Cheng, E. Lustig, K. Wang, S. Fan, Multi-dimensional band structure spectroscopy in the synthetic frequency dimension. *Light Sci. Appl.* **12**, 158 (2023).

57. Cheng, D., Wang, K., and Fan, S. Artificial non-Abelian lattice gauge fields for photons in the synthetic frequency dimension. *Phys. Rev. Lett.* **130**, 083601 (2023).

58. Y. Wang, Y. Liu, J. Zhan, S. Xue, Y. Zheng, R. Zeng, Z. Wu, Z. Wang, Q. Zheng, D. Wang, W. Shi, X. Fu, P. Xu, Y. Wang, Y. Liu, J. Ding, G. Huang, C. Yu, A. Huang, X. Qiang, M. Deng, W. Xu, K. Lu, X. Yang, J. Wu, Large-scale full-programmable quantum walk and its applications. arXiv:2208.13186 [quant.ph] (28 August 2022).

59. R. Konoike, A. Yoshizawa, S. Namiki, K. Ikeda, Quantum walk experiments using a 32 × 32 silicon photonic path-independent insertion loss switch as a stable multiport interferometer, *J. Lightw. Technol.* **40**, 7619–7625 (2022).

60. J. Mower, N. C. Harris, G. R. Steinbrecher, Y. Lahini, D. Englund, High-fidelity quantum state evolution in imperfect photonic integrated circuits. *Phys. Rev. A* **92**, 032322 (2015).
